# Constructing a novel signature and predicting the immune landscape of colon cancer using N6-methylandenosine-related lncRNAs

**DOI:** 10.3389/fgene.2023.906346

**Published:** 2023-06-16

**Authors:** Yongfeng Wang, Dongzhi Zhang, Yuxi Li, Yue Wu, Haizhong Ma, Xianglai Jiang, Liangyin Fu, Guangming Zhang, Haolan Wang, Xingguang Liu, Hui Cai

**Affiliations:** ^1^ The First Clinical Medical College of Lanzhou University, Lanzhou, Gansu, China; ^2^ General Surgery Clinical Medical Center, Gansu Provincial Hospital, Lanzhou, Gansu, China; ^3^ Key Laboratory of Molecular Diagnostics and Precision Medicine for Surgical Oncology in Gansu Province, Gansu Provincial Hospital, Gansu, China; ^4^ NHC Key Laboratory of Diagnosis and Therapy of Gastrointestinal Tumor, Gansu Provincial Hospital, Lanzhou, China

**Keywords:** colon cancer, m6A-related lncRNAs, tumor immune microenvironment, prognostic signatures, immune landscape

## Abstract

**Background:** Colon cancer (CC) is a prevalent malignant tumor that affects people all around the world. In this study, N6-methylandenosine-related long non-coding RNAs (m6A-related lncRNAs) in 473 colon cancers and 41 adjacent tissues of CC patients from The Cancer Genome Atlas (TCGA) were investigated.

**Method:** The Pearson correlation analysis was conducted to examine the m6A-related lncRNAs, and the univariate Cox regression analysis was performed to screen 38 prognostic m6A-related lncRNAs. The least absolute shrinkage and selection operator (LASSO) regression analysis were carried out on 38 prognostic lncRNAs to develop a 14 m6A-related lncRNAs prognostic signature (m6A-LPS) in CC. The availability of the m6A-LPS was evaluated using the Kaplan–Meier and Receiver Operating Characteristic (ROC) curves.

**Results:** Three m6A modification patterns with significantly different N stages, survival time, and immune landscapes were identified. It has been discovered that the m6A-LPS, which is based on 14 m6A-related lncRNAs (*TNFRSF10A-AS1, AC245041.1, AL513550.1, UTAT33, SNHG26, AC092944.1, ITGB1-DT, AL138921.1, AC099850.3, NCBP2-AS1, AL137782.1, AC073896.3, AP006621.2, AC147651.1*), may represent a new, promising biomarker with great potential. It was re-evaluated in terms of survival rate, clinical features, tumor infiltration immune cells, biomarkers related to Immune Checkpoint Inhibitors (ICIs), and chemotherapeutic drug efficacy. The m6A-LPS has been revealed to be a novel potential and promising predictor for evaluating the prognosis of CC patients.

**Conclusion:** This study revealed that the risk signature is a promising predictive indicator that may provide more accurate clinical applications in CC therapeutics and enable effective therapy strategies for clinicians.

## Introduction

Globally, colon cancer is a common malignant tumor in humans that has a high morbidity and mortality rate (Lu and Zhang, 2020; Xiaoyong et al., 2022). A poor prognosis leads to CC posing a serious threat to the health of humans. Most colon cancers are caused by poor dietary habits, age, lifestyle, lack of exercise, and smoking (Xavier and Podolsky, 2007). In recent years, there have been increased studies focusing on the modification of m6A within the epigenetics field. The m6A modification, which transfers the methyl group to the nitrogen-6 position of the adenosine base in RNA, is the most abundant and reversible mRNA epigenetic modification (Zhang et al., 2021a). The m6A methyltransferase, also known as “writers”, installs m6A modification from RNA, while the demethylase, also known as “erasers”, removes m6A modification from RNA (Xu et al., 2021a). After m6A modification, the mature mRNA is recognized by the “reader” when it is exported from the nucleus to the cytoplasm (Zaccara et al., 2019). The “reader” is a binding protein capable of recognizing important chemical signals for m6A modification. As a promising biomarker, m6A-related regulatory factors participate in various biological processes in the occurrence and progression of numerous diseases, especially malignant tumors (Tian et al., 2020). The m6A-related regulatory factors are involved in almost every RNA metabolism and processing step that influence RNA function (Lan et al., 2021).

Long non-coding RNA (lncRNA), a type of transcribed RNA, has various important biological functions (Johnsson et al., 2014). As lncRNA is vital in almost all aspects of biological function, it is indispensable for modulation, especially m6A. Furthermore, lncRNA may also regulate m6A methylation through certain pathways. In addition, m6A modification may affect lncRNA by several regulatory mechanisms. Furthermore, m6A modification may alter the structure of sectional RNA, allowing the corresponding RNA-binding proteins to enter the ambient m6A residues. Moreover, modification of m6A may affect the formation of the RNA-DNA triplex, modulating the binding of RNA to target DNA, and thereby regulating target genomic sites (Ma et al., 2019). It has been reported that m6A-related lncRNAs contribute to improving prognostic risk assessment and the development of individualized therapy decisions in various cancers. For example, Feng et al. (2021) demonstrated the prognostic predictive value of the 6A-LPS, which included four m6A-related lncRNAs, and evaluated the correlation with *PD-L1* in head and neck squamous cell carcinoma. Xu et al. (2021b) elucidated a risk signature composed of 12 m6A-related lncRNAs as promising prediction targets in lung adenocarcinoma prognosis and immune responses. The m6A-related lncRNA signature has been shown to have significant molecular prognostic value in gastric cancer and may improve the development of personalized immunotherapy strategies (Wang et al., 2021). However, the specific mechanism through which m6A-related lncRNAs promote CC occurrence and development remains unknown.

In this study, the expression data of 14,142 lncRNAs and 21 m6A regulators from TCGA were analyzed to identify three m6A modification patterns, as well as develop an m6A-LPS using the LASSO regression analysis. The 14 lncRNAs used to construct the m6A-LPS were reported in CC for the first time. In addition, a nomogram was established to predict the survival time of CC patients. Finally, the relationship with immunotherapy responses was investigated.

## Materials and methods

### Datasets and screening of m6A-related lncRNAs

The transcriptome sequencing results, relevant clinical details, and somatic mutation data of CC patients were collated via TCGA (https://cancergenome.nih.gov/), which embodied mRNA and lncRNA expression data from 473 colon cancers and 41 adjacent tissues of CC patients. The GTF file was downloaded via Ensembl for annotation to distinguish between mRNA and lncRNAs. Furthermore, a list of 21 m6A-related genes was screened based on previous literature, including 8 writers (*RBM15, RBM15B, WTAP, METTL3, METTL14, KIAA1429, CBLL1*, and *ZC3H13*), 2 erasers (*FTO* and *ALKBH5*), and 11 readers (*YTHDC1, YTHDC2, IGF2BP1, IGF2BP2, IGF2BP3, HNRNPC, HNRNPA2B1*, *YTHDF1, YTHDF2, YTHDF3,* and *RBMX*). If the expression level of a lncRNA was related to one or more of these genes, it was identified as an m6A-related lncRNA. The correlation between lncRNA and m6a regulators in the dataset was determined by the Pearson method. The square of correlation coefficient∣*R* ^2^∣>0.5 and *p* < 0.001 were used as the identification criteria for m6A-related lncRNAs.

### Consensus clustering and comparison of immune cell infiltration

Consensus clustering analysis was used to identify distinct modification patterns and cluster the TCGA-COAD samples based on the expression levels of prognostic lncRNAs. Consensus clustering was used to determine the optimal number of clusters. The ConsensusClusterPlus R package, which was the unsupervised clustering method, provided stable visual evidence to estimate the number of unsupervised clusters in a dataset (Wilkerson et al., 2010). In order to ensure the classification was stable, 1,000 repetitions were performed utilizing ConsensuClusterPlus package (Wilkerson et al., 2010). In order to analyze the differences in immune infiltration levels among multiple clusters, 22 immune cell types infiltrating the TCGA-COAD samples were identified using the CIBERSORT package (Wang et al., 2021; Wang et al., 2021; Y et al., 2022). The *p*-value corresponding to the output result is of < 0.05 was considered statistically significant.

### Construction of the m6A-LPS

The single-factor Cox regression was used in conjunction with survival data, and the prognosis-related lncRNAs with *p* < 0.05 were screened using the log-rank test according to the TCGA database. Subsequently, the Lasso regression analysis was carried out on these lncRNAs to develop the m6A-LPS. The risk score for each case was determined based on the expression of the predicted lncRNA multiplied by the coefficient from the LASSO algorithm. The Kaplan–Meier (K-M) method was used for assessing the difference in overall survival (OS). The receiver operating characteristic (ROC) curve was drawn using the R package “survivalROC”. The sensitivity and specificity of the m6A-LPS were examined using the area under the curve (AUC). In order to determine if the m6A- LPS and clinicopathological features served as independent factors for OS, the univariate and multivariate Cox regression analyses were carried out. The principal component analysis (PCA) was used to compare the distribution of high- and low-risk score patients in CC by the “prcomp” function of the “stats” R package.

### Estimation of immune landscape and immunosuppressive molecules with the m6A-LPS

In order to assess the various immune landscapes in the different risk subgroups, the currently acknowledged methods were utilized to identify the infiltration of 22 immune cell types in the TCGA-COAD samples, including TIMER (Li et al., 2017; Wang et al., 2020), CIBER SORT (Chen et al., 2018; Zhang et al., 2020), XCELL (Aran et al., 2017; Aran, 2020), QUANTISEQ (Plattner et al., 2020), MCPcounter (Dienstmann et al., 2019), EPIC (Racle et al., 2017), and CIBERSORT-ABS (Tamminga et al., 2020). A detailed Spearman correlation analysis was performed using the R ggplot2 packages. A *p*-value of < 0.05 was considered statistically significant. The differences of 22 immune cell types were also analyzed by the Wilcoxon test using the R ggpubr package. The differences in the expression levels of biomarkers related to Immune Checkpoint Inhibitors (ICIs) between different subgroups were examined using a violin plot obtained with the R ggpubr package.

### GSEA enrichment analysis

We used GSEA to examine immune function and biological pathways. Functional enrichment analyses via Gene Ontology (GO) and the Kyoto Gene and Genomic Encyclopedia (KEGG) pathway analyses, were conducted by gene set enrichment analysis (GSEA) 4.1.0 (Subramanian et al., 2005; Kanehisa et al., 2017). *p* < 0.05 was considered a significant category.

### Quantitative real-time PCR

In accordance with the instructions of the manufacturer, cDNA synthesis was performed by using the SYBR-Green Mix (Vazyme Biotech Co.,Ltd.). The Real-time PCR (qRT-PCR) analysis was conducted on The LightCycler 480 Real-Time PCR System. The primers used for qRT-PCR were purchased from Servicebio (Wuhan China). The related GAPDH mRNA expression was used as an endogenous control. The primer sequences used in our study were summarized in Table 1.

**TABLE 1:** The primer sequences for qRT-PCR.

Gene	The primer sequences (5′-3′)
AC245041.1-F	GGA​TGT​GCC​ATG​ACT​GCT​TAC​A
AC245041.1-R	ACG​CCA​CTG​CCT​TCT​CAA​ACT
AL513550.1-F	GCA​ACT​CCA​CTT​ACA​GAC​TAC​GGA
AL513550.1-R	GTG​GTG​GAA​CTG​TAT​CTG​CAA​CAA​A
AC099850.3-F	AGT​GGC​AGT​GTT​GCA​ATC​TCG
AC099850.3-R	AAG​GAA​TCT​CTG​AAG​TCC​ATA​GCA​G
AL137782.1-F	GTC​AAT​GAG​CCC​TGA​AGA​ACG​A
AL137782.1-R	GCA​CAT​ATC​AGT​TGC​CTC​CAA​A
AC073896.3-F	GAA​ACC​CTG​AGA​CAA​CCA​TAC​C
AC073896.3-R	TCT​CCT​GAC​TTC​GTG​ATC​CG
AP006621.2-F	GAT​GCG​GAA​CCC​ATA​GAT​CCT
AP006621.2-R	CGT​CTT​AGC​GGC​TGT​CAC​TTA​CT
AC147651.1-F	CAT​GGA​AGC​TCC​GGG​TTT​C
AC147651.1-R	CTC​CTC​CTT​GGT​GTC​CCA​GAT​T
SNHG26-F	GGT​CTG​GCG​CTT​GAA​AGA​ATC
SNHG26-R	AGG​GGG​CCT​TCT​AGT​CAT​GG
AL138921.1-F	CCT​GCC​ATC​TAT​CCT​CCA​ACT​C
AL138921.1-R	GAA​CTA​CTG​TGC​TGG​CAA​ACC​C
AC092944.1-F	TCC​AGA​TCA​CCA​CAC​CAC​ATC
AC092944.1-R	ACA​AAC​TGC​CCG​CAC​CTT​A
TNFRSF10A-AS1 (1)-F	ACA​TTT​GTT​TAG​GAT​GAG​AGC​TGC
TNFRSF10A-AS1 (1)-R	GGC​CGT​CCA​GTA​AGC​TAA​GGT
ITGB1-DT-F	ATA​ATT​GGT​CCG​TGC​CTG​ATT​T
ITGB1-DT-R	ACA​GTG​CTT​GAC​GGT​GGT​GTT​A
NCBP2-AS1-F	GTG​GGT​AGG​ATC​ACT​TAG​GCT​CA
NCBP2-AS1-R	CAT​TGT​GGT​CCG​CTT​CTC​TG

### Statistical analysis

Gene expression and risk scores between subgroups were compared using the Wilcox test. The Kaplan–Meier (K-M) method was used for assessing the difference in OS among three subgroups. An assessment of linear correlation between two random variables was performed using the Pearson correlation coefficient. All statistical analyses were performed using R (version 3.6.2), GraphPad Prism 9 and Perl software in the present experiment. All analyses performed were bilateral, with *p* < 0.05 being statistically significant.

## Results

### Determining m6A-related lncRNAs in CC patients

**FIGURE 1:**
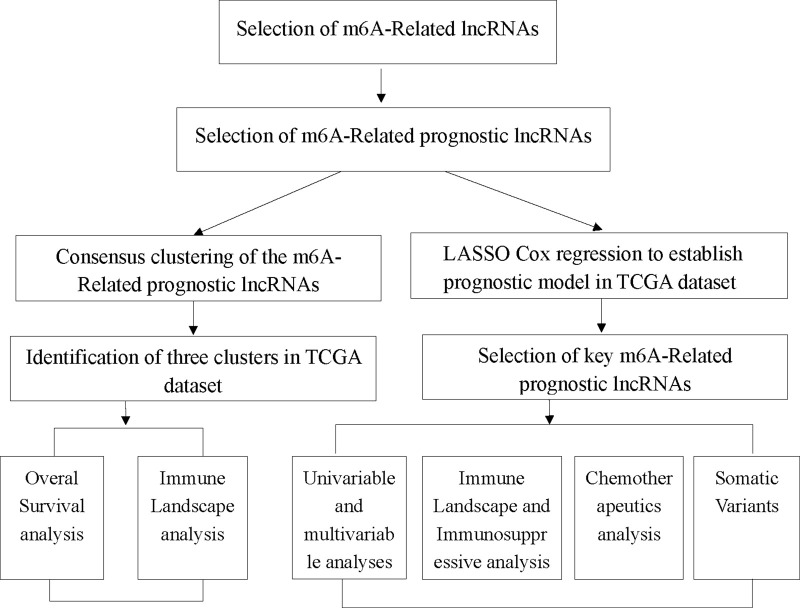
Study flow chart.

**FIGURE 2:**
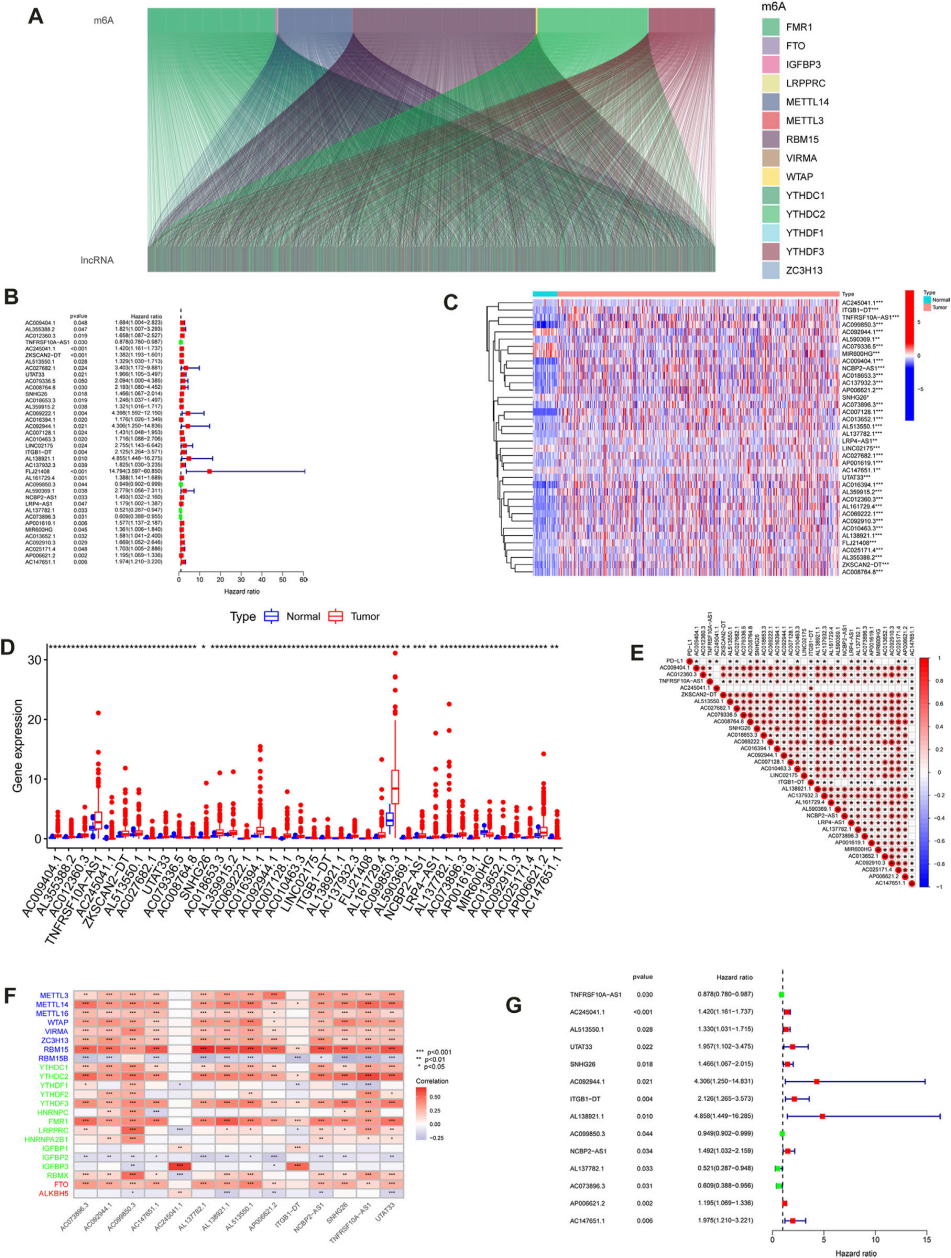
Identification of m6A-related lncRNAs in CC. **(A)** Sankey relational diagram for 21 m6A genes and m6A-related lncRNAs. **(B)** Univariate Cox regression analysis forest map of the 38 m6A-Related prognostic lncRNAs. Heat map **(C)** and box plot **(D)** of the differential expression of the 38 m6A-Related prognostic lncRNAs. **(E)** The correlation among expression of the m6A-related prognostic lncRNAs and PD-L1. **(F)** Heatmap for the correlations between 21 m6A genes and the 14 prognostic m6A-related lncRNAs. **(G)** Univariate Cox regression analysis for 14 m6A-related prognostic lncRNAs in the m6A-LPS. (***p < 0.001; **p < 0.01; *p < 0.05)

The workflow for the m6A-LPS analysis is illustrated in Figure 1. Initially, the expression data of 14,142 lncRNAs and 21 m6A regulators from TCGA, as well as the survival status, and clinical data, were identified and obtained. A total of 1,234 lncRNAs were identified by determining the Pearson coefficient of the lncRNA-mRNA co-expression analysis (Figure 2A; Supplementary Table S1). As shown in Figure 2B; Supplementary Table S2, 38 prognostic m6A-related lncRNAs were filtered through a univariate Cox regression analysis. The differences in expression of the 38 prognostic lncRNAs in CC and normal samples were analyzed using the Wilcoxon test. The differential expression of 38 lncRNA heatmaps (Figure 2C) and boxplots (Figure 2D) were plotted using the “pheatmap” and “ggpot2” packages, respectively. The correlations between the 38 prognostic lncRNAs and PD-L1 in TCGA were examined using Spearman’s method, as displayed in Figure 2E

### Consensus clustering and comparison of immune cell infiltration

**FIGURE 3:**
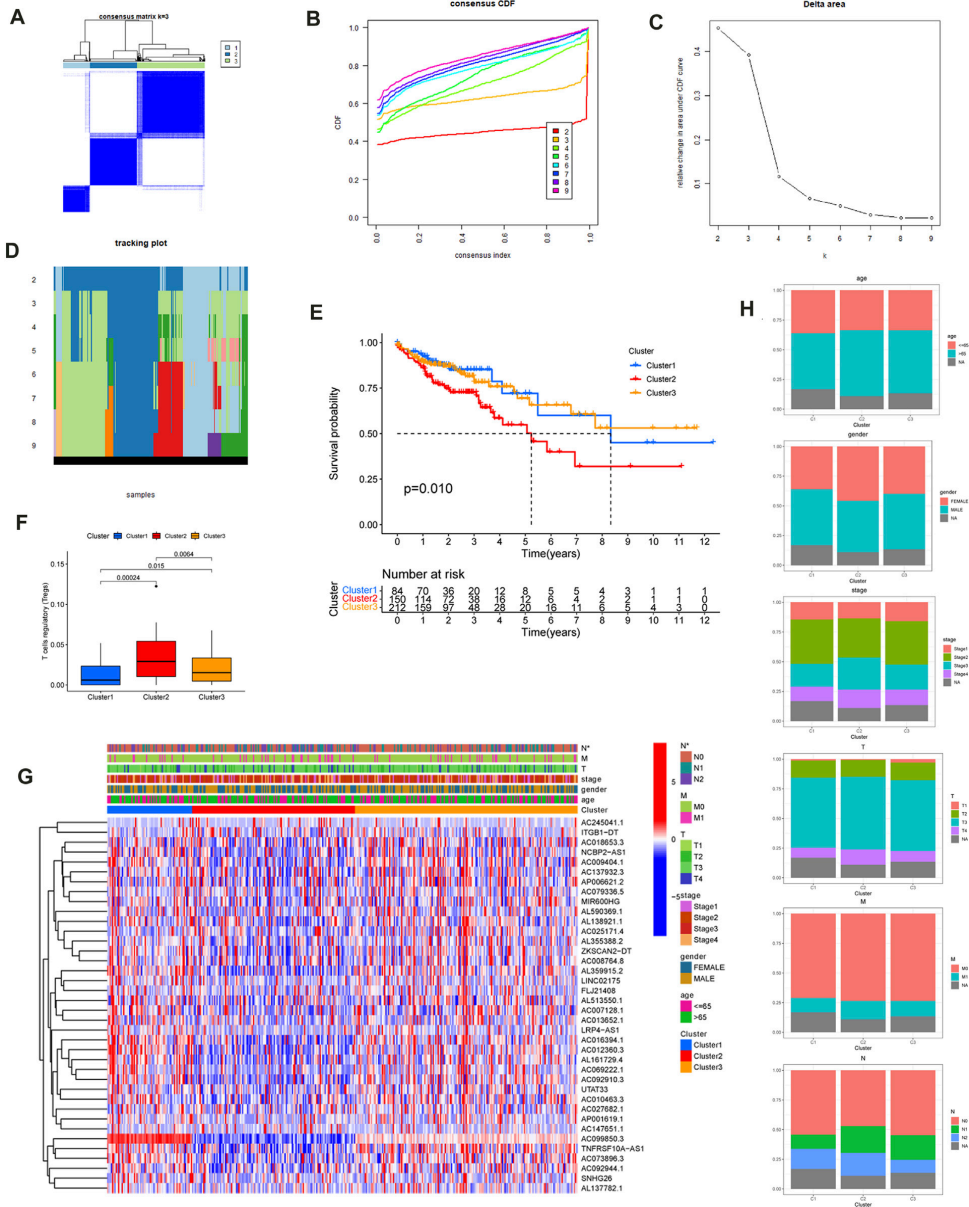
Consensus Clustering of the m6A-Related prognostic lncRNAs and Comparison of Immune Cell Infiltration. **(A)** The consensus clustering matrix at k = 3 and their correlation area. The rows and columns of the matrix represent samples. The values of the consistency matrix are shown in white to dark blue from 0 to 1, which represents the degree of consensus. **(B)** Consensus clustering cumulative distribution function (CDF) for k = 2 to 9. **(C)** The relative variation of the area under the CDF curve that k from 2 to 9. Delta area curve of consensus clustering, indicating the relative change in area under the CDF curve for each category number k compared with k–1. The horizontal axis represents the category number k and the vertical axis represents the relative change in area under CDF curve. **(D)** Tracking plot for k from 2 to 9. **(E)** Kaplan–Meier survival curves of COAD for three clusters. **(F)** The T cells regulatory (Tregs) differed significantly among three clusters. **(G)**The heatmap of the expression of the m6A-Related prognostic lncRNAs for three clusters. (***p < 0.001; **p < 0.01; *p < 0.05). **(H)** age, gender, T stage, N stage, M stage, and clinical stage distributions for three clusters.

Consensus cluster analysis was used to further explore the expression characteristics of m6A-related lncRNAs in the TCGA-COAD samples. The TCGA-COAD cohort was clustered into k subtypes (k = 2–9) based on the 38 prognostic lncRNA expression levels with the R package ConsensusClusterPlus (Figures 3A–D; Supplementary Figure S1). The proportion of ambiguous clustering measurements and the similarity in expression levels we measured for the m6A-related lncRNAs shared by TCGA ultimately determined k = 3 to have the best cluster stability. There was also the least crossover between CC samples, when the consistency matrix with a k value of 3 was selected.

In order to explore the clinical application value among the three clusters, the relationship between cluster and clinical features was evaluated using the Chi-square test, and the obtained heatmap is displayed in Figure 3G Among the three clusters, there was a significant difference in the N stage and, according to the Kaplan–Meier method (*p* = 0.010) (Figure 3E), a clear difference in survival time. The intracluster proportions for the three clusters were then analyzed based on age, gender, T, N, M, and clinical stage as shown in Figure 3H Subsequently, the CIBERSORT algorithm was used to analyze the infiltration of 22 immune cell types among three clusters. It was found that the Tregs differed significantly among the three clusters (Supplementary Figure S2). The T cells regulatory (Tregs) differed significantly among three clusters (Figure 3F). Furthermore, cluster3 displayed a greater number of Mast cells resting compared with cluster1. The NK cells resting, CD4 T cells memory activated, CD8 T cells, T cells gamma delta, and T cells regulatory differed significantly between cluster1 and cluster2. The T cells CD4 memory activated, NK cells resting, T cells gamma delta, and T cells regulatory differed significantly between cluster3 and cluster2.

### Construction of the m6A-LPS

**FIGURE 4:**
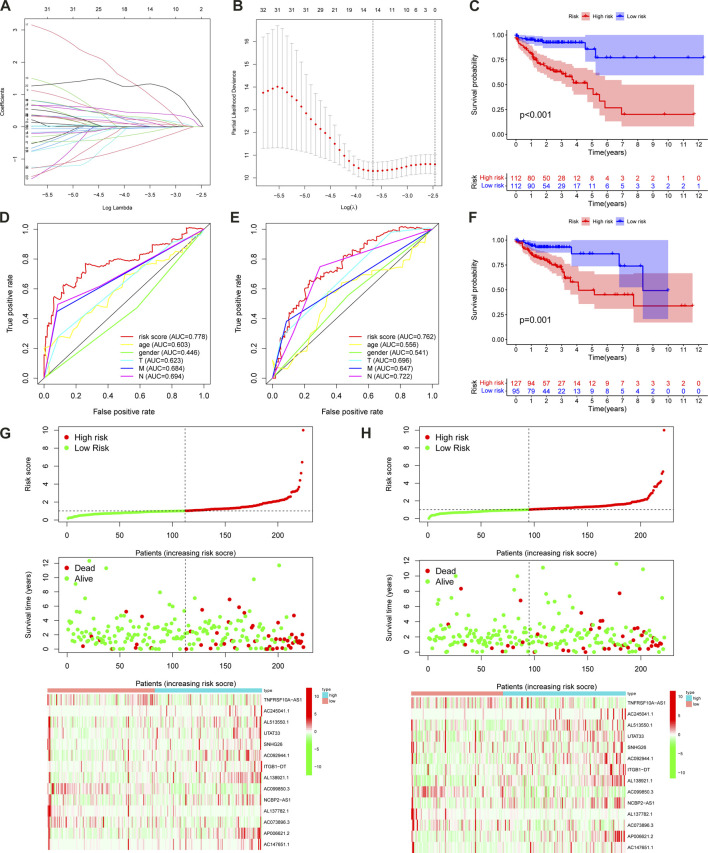
The m6A-LPS predicts overall survival in patients with CC. LASSO regression was performed, calculating the minimum criteria **(A,B)**. The patients in the high-risk cohort had significantly shorter OS than those in the low-risk cohort in the training **(C)** and test sets **(F)**; ROC curves of the m6A-LPS and clinical features for prediction of 3-year OS in the training **(D)** and test sets **(E)**; risk score distribution, the distribution of survival time and survival status, and the heatmap of the expression of 14 m6A-related prognostic lncRNAs in the training **(G)** and test sets **(H)**.

The LASSO regression analysis was performed on 38 prognostic lncRNAs to develop a 14 m6A-LPS in TCGA dataset (Figures 4A, B; Supplementary Table 3). The correlations between the 14 prognostic lncRNAs and the m6A genes in TCGA were displayed in Figure 2F As shown in Figure 2G; Table 2, forest maps corresponding to these 14 prognostic lncRNAs were drawn, with the prognostic favorable factors *TNFRSF10A-AS1, AC099850.3, AL137782.1,* and *AC073896.3* among them. The rest, on the other hand, were classified as prognostic unfavorable factors, and their over-expression might reduce survival. The Kaplan–Meier method was used to determine the prognostic value of the 14 hub lncRNAs in CC, with the median expression being the cutoff value. As displayed in Supplementary Figure S3, the four of the 14 hub lncRNAs (*AC092944.1, AL138921.1, SNHG26*, and *TNFRSF10A-AS1*) were identified using the Kaplan Meier-plotter (*p* < 0.05).

**TABLE 2:** Univariate Cox regression analysis of the 14 m6A-related prognostic lncRNAs.

Gene	HR	HR.95L	HR.95H	*p*-value
TNFRSF10A-AS1	0.877764	0.780347	0.987343	0.029842
AC245041.1	1.420121	1.16086	1.737284	0.000649
AL513550.1	1.32972	1.031234	1.7146	0.028014
UTAT33	1.956639	1.101711	3.474992	0.021992
SNHG26	1.465938	1.066651	2.014693	0.018389
AC092944.1	4.305508	1.249897	14.83114	0.020698
ITGB1-DT	2.126028	1.265159	3.572669	0.004398
AL138921.1	4.858409	1.449434	16.28507	0.010424
AC099850.3	0.949232	0.902233	0.998679	0.044325
NCBP2-AS1	1.492425	1.03152	2.15927	0.033617
AL137782.1	0.521446	0.286828	0.947978	0.032749
AC073896.3	0.609172	0.38826	0.955779	0.031024
AP006621.2	1.195053	1.06858	1.336495	0.001795
AC147651.1	1.974547	1.210404	3.221101	0.006435

**FIGURE 5:**
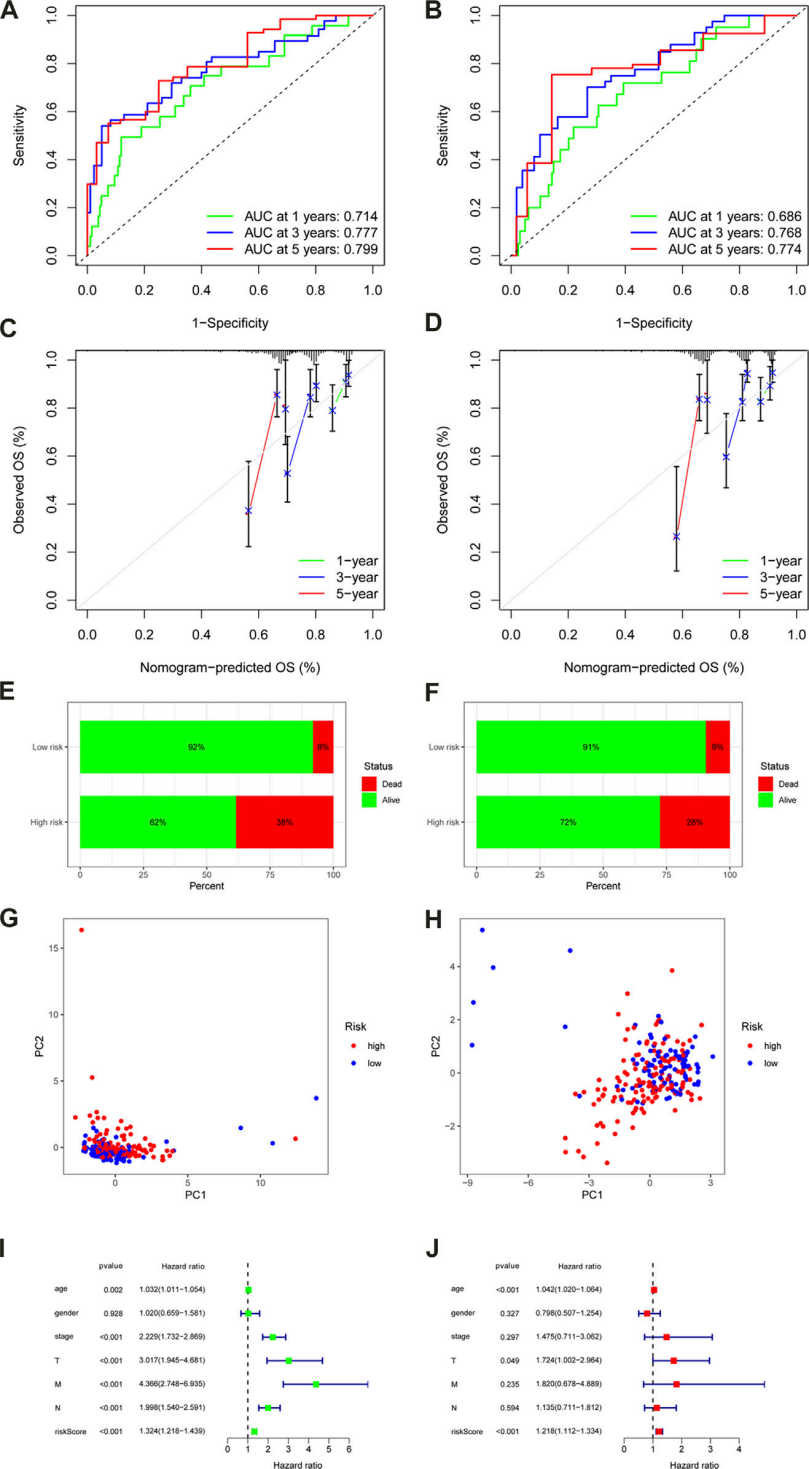
ROC curves of the m6A-LPS to predict 1-, 3-, and 5-year survival in the training **(A)** and test sets **(B)**. A calibration plot of the m6A-LPS to predict 1-, 3-, and 5-year survival in the training **(C)** and test sets **(D)**. Mortality rates of the low- and high-risk subgroups in the training **(E)** and test sets **(F)**. Principal component analysis (PCA) plot in the training **(G)** and test sets **(H)**. Univariable **(I)**and multivariable **(J)** analyses for the risk score and other clinical features.

We obtained the risk score of each case; risk score = (−0.02102 × *TNFRSF10A-AS1* expression) + (0.259351 × *AC245041.1* expression) + (0.19614 × *AL513550.1* expression) + (0.21851 × *UTAT33* expression) + (0.075165 × *SNHG26* expression) + (1.187719 × *AC092944.1* expression) + (0.433276 × *ITGB1-DT* expression) + (1.296707 × *AL138921.1* expression) + (−0.04355 × AC099850.3 expression) + (0.034475 × *NCBP2-AS1* expression) + (−0.53815 × *AL137782.1* expression) + (−0.20653 × *AC073896.3* expression) + (0.09041 × *AP006621.2* expression) + (0.299669 × *AC147651.1* expression). In order to validate the accuracy of the m6A-LPS, all cases were randomly separated into either a training set or test set. Furthermore, all cases in the TCGA-COAD cohort were separated into high- and low-risk subgroups in every set based on the median risk score. The K-M analysis revealed that the OS of high-risk patients declined compared to the low-risk patients in the training set (*p* < 0.001) (Figure 4C), which was further validated by the results of the test set (*p* = 0.001) (Figure 4F). Finally, the ROC curve revealed that the m6A-LPS exhibited a relatively decent predictive value in both the training and test sets in predicting a 3-year OS of CC in comparison to other clinical factors, as shown in Figures 4D, E. Figures 4G, H displays the risk score distribution, survival status, survival time, and expression heatmap based on the m6A-LPS in both the training and test sets. The ROC analysis revealed that the AUCs of the m6A-LPS at 1-, 3-, 5-year was 0.714, 0.777, 0.799 in the training set, respectively (Figure 5A); and that the AUCs of the m6A-LPS at 1-, 3-, 5-year was 0.686, 0.768, 0.774 in the test set (Figure 5B), respectively. The calibration curves for the probability of OS at 1, 3, 5 year showed good consistency between the actual observation and the m6A-LPS prediction (Figures 5C, D). According to Figures 5E, F, the mortality rate of the high-risk patients was higher in the training and test sets than in the low-risk patients. Following that, PCA indicated that the training (Figure 5G) and test sets (Figure 5H) should be distributed in two directions.

### An independent prognostic analysis and construction of an m6A-LPS-based nomogram

**FIGURE 6:**
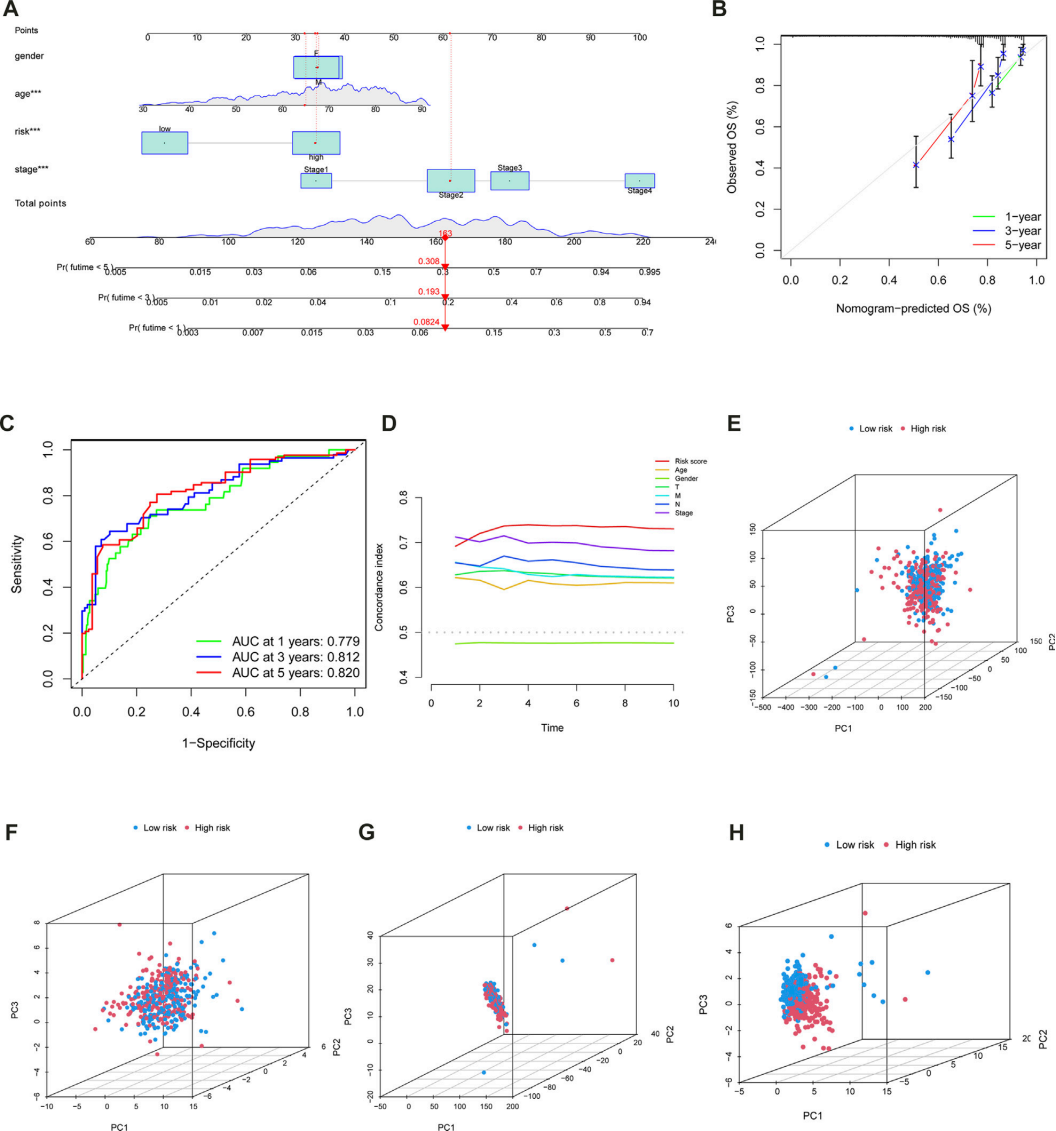
**(A)** Nomogram incorporated with the m6A-LPS and clinical features for prediction of 1-, 3- and 5- years OS in patients with CC. **(B)** Calibration plot of the nomogram to predict 1-, 3-, and 5-year survival. **(C)** ROC curves of the nomogram to predict 1-, 3-, and 5-year survival.f of m6A-LPS for the patients divided by each clinical characteristic. **(D)** The concordance of risk score and other clinical indicators. PCA was used to identify the distinct patterns of m6A distribution on expression profiles of the whole gene **(E)**, 21 m6A genes **(F)**, m6A-related lncRNAs **(G)**, and 14 hub lncRNAs of the m6A-LPS **(H)**.

The univariate and multivariate Cox regression analyses were conducted to confirm whether the m6A-LPS and clinicopathological features had independent prognostic characteristics for patients with CC in the TCGA-COAD cohort. The univariate Cox regression analyses revealed that the m6A-LPS, age, clinical stage, T, M, and N were all significantly related to OS (Figure 5I). Furthermore, the findings of the multivariate Cox analysis indicated that both m6A- LPS, and T were independent predictors for determining the OS of CC patients in the TCGA-COAD cohort (Figure 5J). As an applicable quantitative tool in the clinic, a nomogram containing m6A- LPS with clinical features was established to evaluate the life expectancy of CC patients in the TCGA-COAD cohort (Figure 6A). The total score for each case was obtained by calculating the points of every parameter to predict the 1-year, 3-year, and 5-year OS among CC patients. The calibration curves for the probability of OS at 1, 3, 5 year showed good consistency between the actual observation and the nomogram prediction (Figure 6B). Finally, as shown in Figure 6C, the ROC curve revealed that the m6A-LPS-based nomogram had relatively decent predictive value in predicting 1-year (AUCs = 0.779), 3-year (AUCs = 0.812) and 5-year (AUCs = 0.820) OS.

### PCA confirms the ability of the m6A-LPS to group

To validate risk models, PCA was also applied, and the results were displayed using the R software’s “scatterplot3D” tools. PCA was used to identify the distinct patterns of m6A distribution on expression profiles of the whole gene (Figure 6E), 21 m6A genes (Figure 6F), m6A-related lncRNAs(Figure 6G), and 14 hub lncRNAs of the m6A-LPS (Figure 6H). It was clear from the m6A-LPS results that there were more noticeable differences in distributions between the low- and high-risk groups than from the other three approaches. Based on the m6A-LPS, we intuitively observed that CC patients were effectively divided into two subgroups. The risk score’s concordance index increased over time, outpacing the concordance index of other clinical indicators (Figure 6D). In light of these findings, the m6A-LPS may be a better predictor of the prognosis of CC.

### Clinical correlation analysis

**FIGURE 7:**
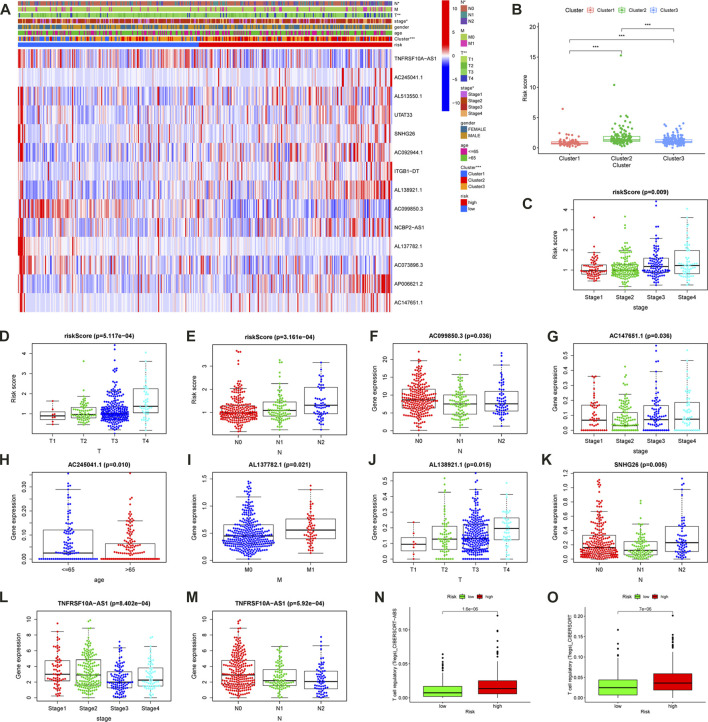
Clinical Application Value of the m6A- LPS. **(A)** The heatmap of m6A-related gene expression, cluster, clinical features, and m6A- LPS. The box plots along with the scatter plots showed that cluster **(B)**, clinical stage**(C)**, T stage**(D)**, and N stage **(E)** were significantly associated with the risk score. **(F–M)** Correlations between 14 hub lncRNAs and clinical features. **(N,O)** The difference of Treg cell infiltration in high and low risk groups.

**FIGURE 8:**
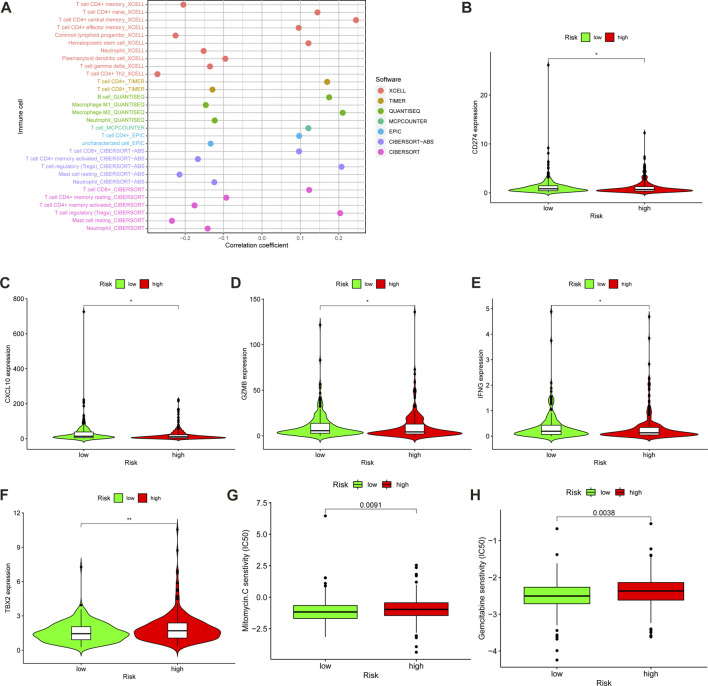
Assessment of Tumor-Infiltrating Cells and Immunosuppressed Molecules by the m6A-LPS. **(A)** The bubble plot visualized the correlation between the m6A-LPS and tumor infiltration immune cells by Spearman correlation analysis. Box-Violin plots visualized the correlation between the m6A-LPS and immune-checkpoint-relevant genes, *CD274* **(B)**, *CXCL10* **(C)**, *GZMB* **(D)**, *IFNG* **(E)**, and *TBX2* **(F)**; *p < 0.05, ** *p* < 0.01, and *** *p* < 0.001. Boxplots evaluating the response to the mitomycin **(G)** and gemcitabine **(H)** chemotherapeutic between high-and low-risk patients.

In order to explore the clinical practice of the m6A- LPS, the chi-square test was used to investigate the relationship between the m6A-LPS and clinical features. The heatmap from the resulting diagram is displayed in Figure 8A. The cluster, T, N, and clinical stages were all significantly different in the various risk-subgroups. Following that, the Wilcoxon or Kruskal-Wallis tests were used to examine the relationships between 14 hub lncRNAs and clinical features (Figure 7B—M; Supplementary Table 4). *AC147651.1*, *TNFRSF10A-AS1,* and the m6A- LPS were significantly linked to the clinical stage; *AL138921.1* and the m6A- LPS were significantly linked to the T stage; *AC099850.3-N*, *SNHG26*, *TNFRSF10A-AS1,* and the m6A- LPS were significantly linked to the N stage; *AC245041.1* was significantly linked to age; *AL137782.1* was significantly associated linked to the M stage.

### Estimation of the immune landscape and immunosuppressive molecules with the m6A-LPS

A detailed correlation analysis using the Spearman method was performed to determine the correlation between the m6A-LPS and tumor-infiltrating immune cells based on the cohort data from TCGA-COAD, with the results shown in a bubble plot (Figure 8A). The results indicated that the m6A-LPS had a negative relation with neutrophils, plasmacytoid dendritic cells, and resting mast cells, and a positive relation with regulatory T cells, monocytes, B cells, and CD8^+^ T cells (Supplementary Figure 5; Supplementary Table 5). The results were similar to consensus clustering analysis. The regulatory T cells are highly expressed in both cluster 2 and high-risk groups, and both have the worse prognosis (Figures 7N, O). As ICIs were recommended for treating CC in clinics, the differences in ICI-related biomarkers between the two subgroups in the TCGA-COAD cohort were investigated. The Box-Violin plots revealed that ICI-related biomarkers, such as *CD274* (*PD-L1*), *CXCL10*, *GZMB*, and *IFNG*, were upregulated in high-risk patients in the TCGA-COAD cohort (Figures 8B–F), whereas *TBX2* was downregulated.

### Investigation of the correlation between the m6A-LPS and chemotherapeutics

In order to evaluate the m6A-LPS in clinical practice for treating CC, the therapeutic response was estimated by calculating the half-maximal inhibitory concentration (IC_50_) of the standard chemotherapeutic drugs for each sample. IC_50_ of chemotherapeutics was obtained from the Genomics of Drug Sensitivity in Cancer (GDSC) website in TCGA. The differences in the IC_50_ between the high- and low-risk subgroups were examined using the Wilcoxon signed-rank test. The results were displayed in a box plot obtained utilizing the R packages ggpubr, pRRophetic, and ggplot2. The box plot revealed a positive association between a high-risk score and a higher IC_50_ of mitomycin (*p* = 0.0091) (Figure 8G) and gemcitabine (*p* = 0.0038) (Figure 8H), suggesting that the m6A-LPS has potential as a predictor in CC patients for chemosensitivity.

### Exploration of the correlation between the m6A-LPS and somatic variants

**FIGURE 9:**
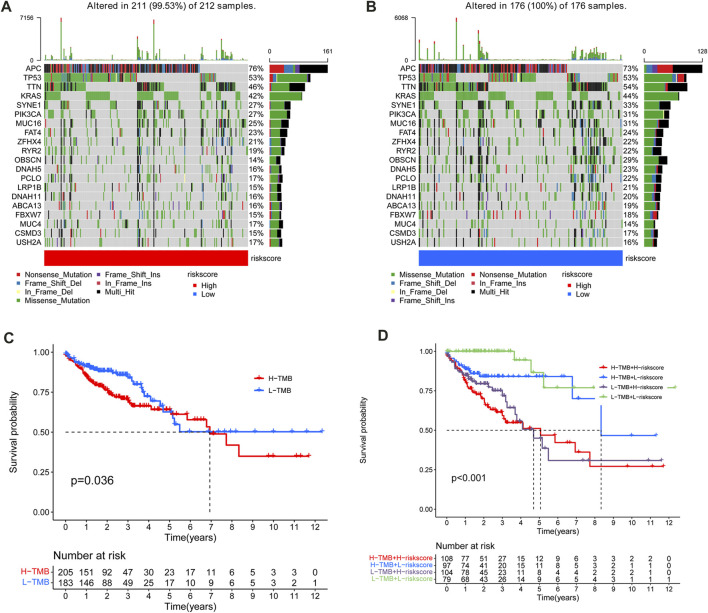
Mutation frequency of the top 20 genes with the most frequent mutations in the training **(A)** and test sets **(B)**. **(C)** Kaplan–Meier curves for patients with high and low TMB subgroups in the TCGA cohort. **(D)**Survival analysis of distinct groups stratified by both TMB and risk score.

The maftools R package was utilized to explore the differences between the two subgroups in tumor mutation burden (TMB) in the TCGA-COAD cohort. Figures 9A, B showed the first 20 most frequently mutated genes in the high- and low-risk subgroups. It indicates that the low-risk subgroup has a more extensive TMB than the high-risk subgroup. Then, all cases in the TCGA-COAD cohort were separated into high TMB and low TMB subgroups according to the cutoff of TMB. The K-M analysis revealed that the OS of the high TMB subgroup declined compared to the low TMB subgroup (*p* = 0.036) (Figure 9C). Following that, the synergistic effect of TMB and risk score in the prognostic stratification of CC patients was assessed. The survival curve of TMB combined with risk score showed that there was a clear difference in survival time among the four subgroups (*p* < 0.001), including high TMB & high-risk score, high TMB & low-risk score, low TMB & low-risk score, and Low TMB & high-risk score (Figure 9D).

### GSEA enrichment analysis

**FIGURE 10:**
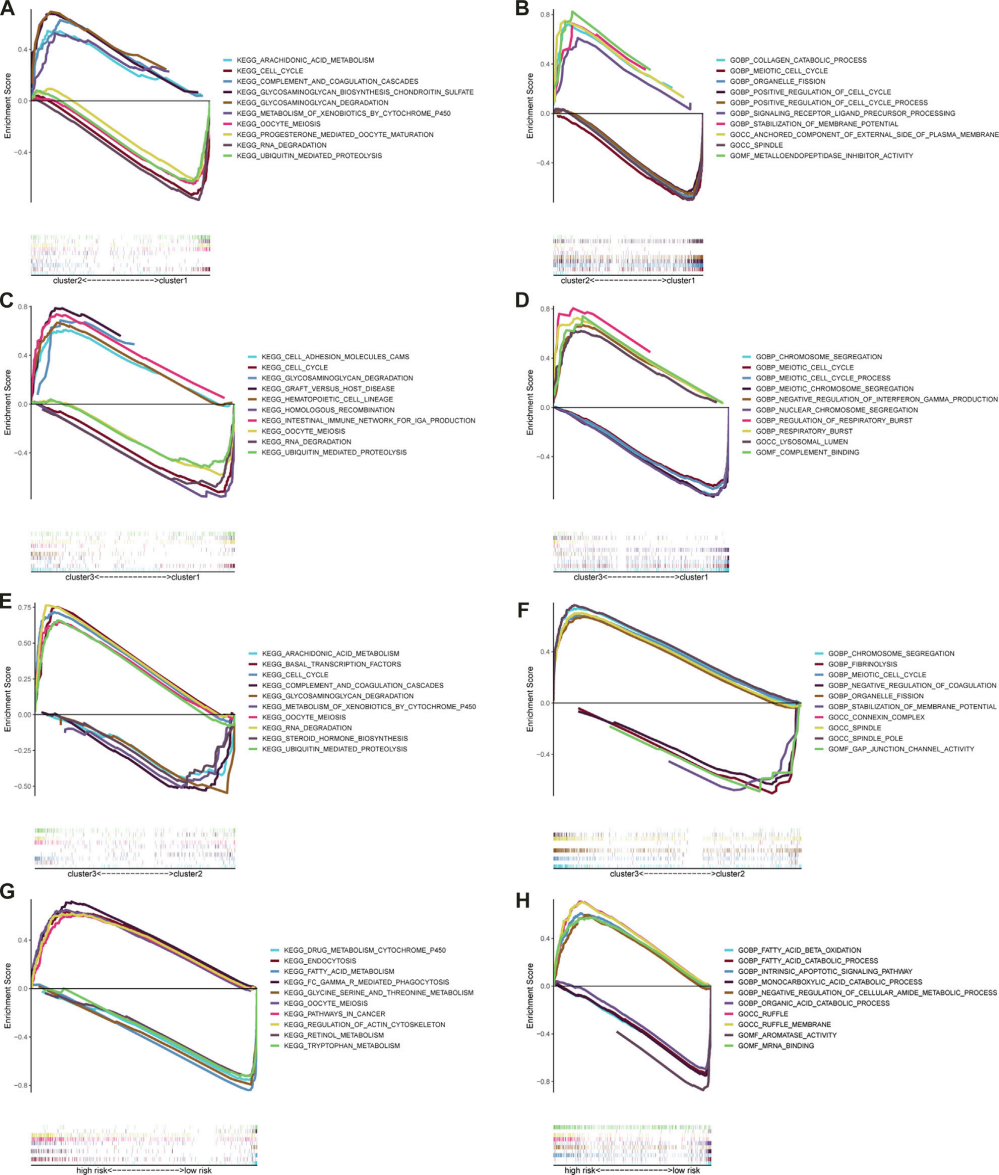
Significantly enriched GO terms and KEGG pathways via GSEA. The top five most relevant items GO terms **(A)** and KEGG pathways **(B)** between the cluster2 and cluster1; The top five most relevant items GO terms **(C)** and KEGG pathways **(D)** between the cluster3 and cluster1; The top five most relevant items GO terms **(E)** and KEGG pathways **(F)** between the cluster3 and cluster2; The top five most relevant items GO terms **(G)** and KEGG pathways **(H)** between the high- and low-risk subgroups.

GSEA was applied to predict the potential GO and KEGG pathways, and the top 5 most relevant items were selected. Figures 10A–F showed the potential functions and access among the three clusters. Following that, GSEA was applied to predict the potential GO and KEGG pathways in the high- and low-risk subgroups. With regard to KEGG pathway analysis as shown in Figure 10G, we found that the following pathways were active in high-risk subgroups: “oocyte meiosis”, “pathways in cancer”, “regulation of actin cytoskeleton”; and that the following pathways were active in low-risk: “drug metabolism cytochrome p450”, “retinol metabolism”, “glycine serine and threonine metabolism”, “tryptophan metabolism”, “fatty acid metabolism”. In addition, the results of GO analysis (Figure 10H) indicated that “negative regulation of cellular amide metabolic process,” and “intrinsic apoptotic signaling pathway,” were mainly enriched in high-risk subgroups, and that “monocarboxylic acid catabolic process,” “organic acid catabolic process,” “aromatase activity,” “fatty acid catabolic process,” and “fatty acid beta-oxidation” were mainly enriched in low-risk subgroups. The results revealed the differentially enriched pathway and biological processes were mostly related to tumor progression and metabolism.

### Validation of survival predictive ability of the m6A-LPS

**FIGURE 11:**
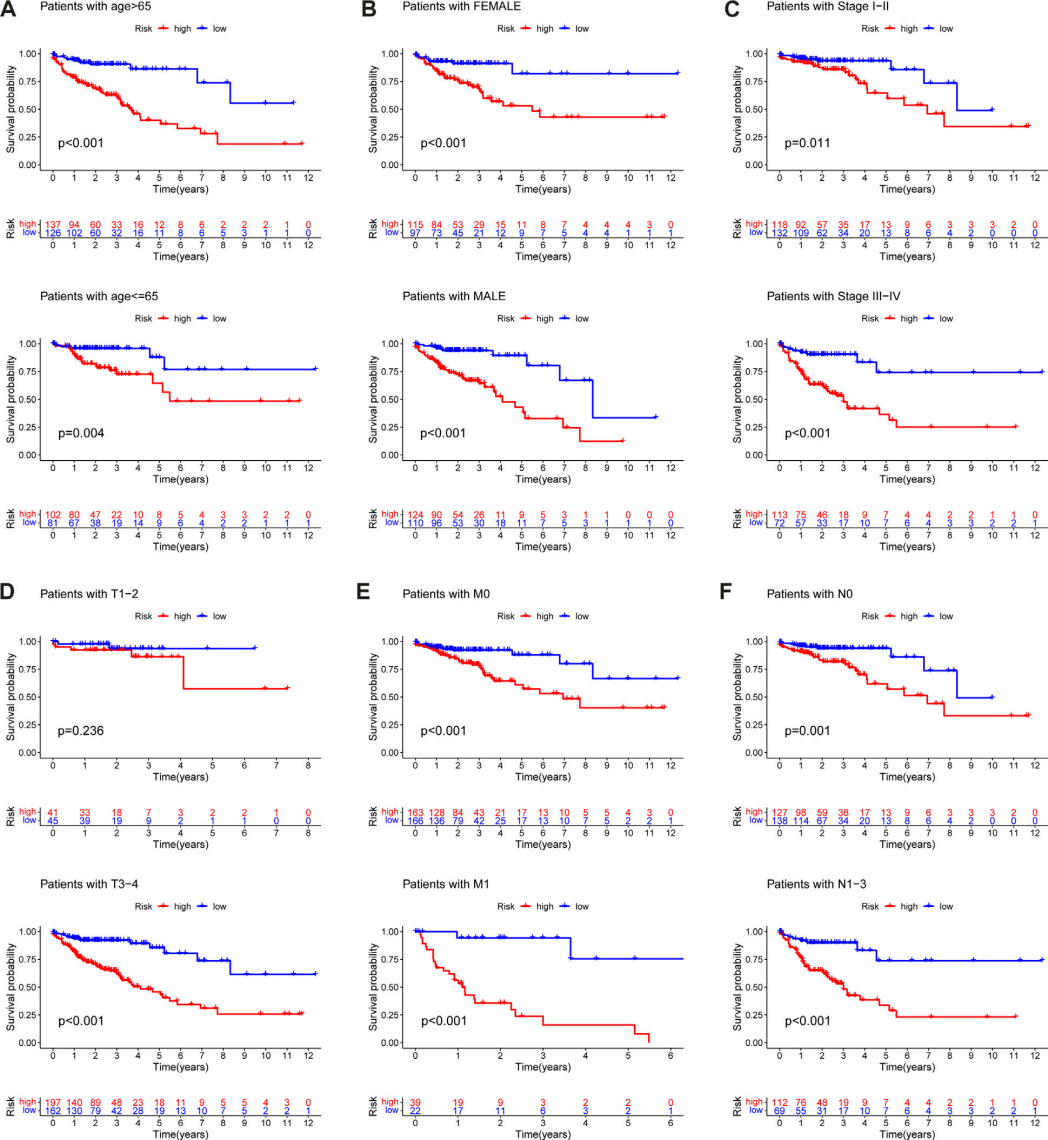
Kaplan–Meier curves for the prognostic value of m6A-LPS for the patients divided by each clinical characteristic. **(A)** age, **(B)** gender, **(C)** stage **(D)** T, **(E)** M, **(F)** N.

The data of 452 CC patients in the TCGA-COAD cohort were examined. In addition, a K-M curve was drawn to demonstrate the correlation between clinicopathologic characteristics and prognosis. The results indicated that age, T, N, M, and clinical stage were statistically significant for prognosis (Supplementary Figure S4). Following that, a stratified analysis for CC patients was performed, which revealed that the m6A-LPS, as a dependable prognostic indicator, had a robust predictive ability for the survival of CC patients (Figure 11A—F). However, the m6A-LPS was not able to independently estimate the survival of CC patients in the T1-2 (Figure 11D) subgroups.

### Experimental validation

**FIGURE 12:**
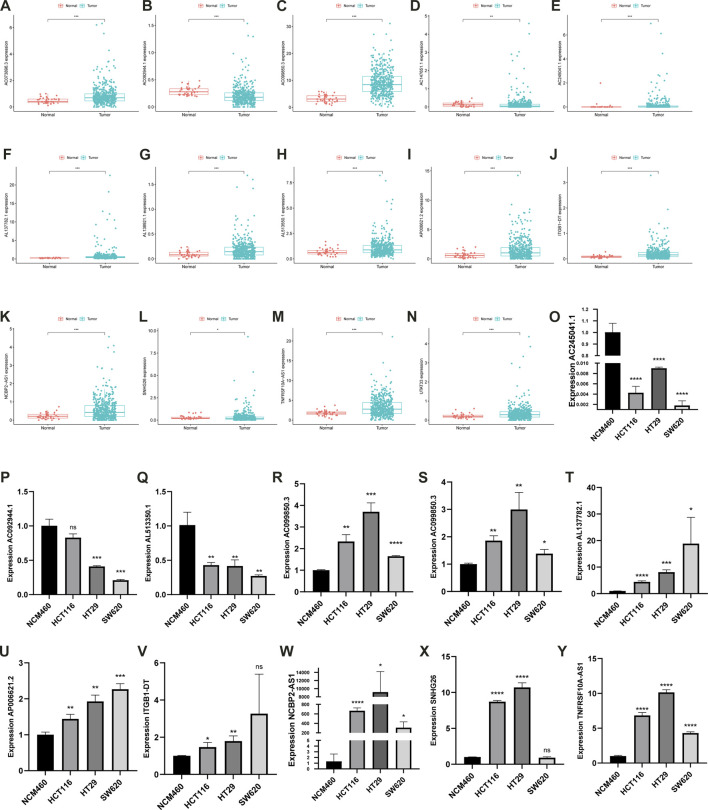
The bar plot of 14 prognostic lncRNAs in normal and tumor tissues **(A–N)**. The expression characteristics of the *AC092944.1, AC245041.1, AL513550.1, AC073896.3, AC099850.3, AL137782.1, AP006621.2, ITGB1-DT, NCBP2-AS1, SNHG26, TNFRSF10A-AS1* in multiple types of CC cell lines **(O–Y)**.

The expression of the 14 prognostic lncRNAs significantly differed in CC and normal samples (*p* < 0.05, Figures 12A–N) in the TCGA-COAD cohort. The expression levels of 11 prognostic lncRNAs were investigated in 3 CC cell lines (HCT116, HT29, SW620), normal colon epithelial cells (NCM460) as control (Figures 12O–Y). The results showed that *AC073896.3, AC099850.3, AL137782.1, AP006621.2, ITGB1-DT, NCBP2-AS1, SNHG26*, and *TNFRSF10A-AS1* were significantly increased in CC cell lines, compared with NCM460. *AC092944.1, AC245041.1,* and *AL513550.1* were significantly decreased in CC cell lines cell line, compared with NCM460.

## Discussion

LncRNAs have been shown to promote or inhibit protein-coding genes in a number of physiological and pathological processes (Wilusz et al., 2009; Ma et al., 2017; Zhang et al., 2019). Increasing evidence suggests that m6A-related genes can regulate the incidence and progression of a variety of malignant tumors by modifying specific lncRNAs. Furthermore, lncRNAs could be used as competitive endogenous RNAs to regulate tumor invasive progression by targeting m6A-related genes.

Recently, several studies reported that the m6A-associated regulators were dysregulated in CC, as well as being involved in the initiation and progression of the disease. Ni et al. (2019) identified a novel negative functional loop, the lncRNA *GAS5-YAP-YTHDF3* axis, in a clinical study. Compared to the adjacent tissues, the expression of *GAS5* was lower in CRC tissues. In CRC patients, the high expression levels of *YAP* and *YTHDF3* were linked to the low expression of *GAS5*. These findings offer a novel therapeutic option for CRC patients. According to Yang et al. (2020), *METTL14* was downregulated in CRC and the low expression of *METTL14* may be associated with the proliferation and invasion in CRC. Moreover, it was discovered that lncRNA *XIST* was a downstream target of *METTL14*, and that the expression of *XIST* was negatively correlated with *METTL14* and *YTHDF2*. The lncRNA *RP11* is upregulated in clinical settings and is regulated by m6A methylation. Furthermore, m6A-induced lncRNA *RP11* can promote CRC cell metastasis and proliferation through the upregulation of Zeb1 (Wu et al., 2019).

The majority of previously published studies focused on the intrinsic carcinogenic pathways of CC. However, how lncRNA interacts with m6A regulators in carcinogenesis and progression of CC, as well as the overall patterns of the m6A-related lncRNA in CC, remain unknown. Therefore, in-depth studies of the m6A-related lncRNA will contribute to the identification of therapeutic biomarkers that have clinical prognostic value and the development of more effective therapeutic strategies. In the present study, we identified the m6A-related lncRNAs using correlation analysis implemented by the Pearson method from TCGA. The TCGA confirmed 38 m6A-related lncRNAs related to the prognosis, and three m6A modification patterns with significantly different N stages, survival time, and immune landscape were identified according to these lncRNAs. Additionally, 14 of 38 prognostic lncRNAs were applied to develop an m6A-related lncRNA model to predict the OS of CC patients. In further analysis, the overall survival, cluster, T, N, and clinical stage significantly differed in the different risk-subgroups in further analysis. Notably, the ROC curve revealed that the m6A-LPS exhibited a relatively decent predictive value in both the training and test sets in predicting a 3-year OS of CC in comparison to other clinical factors. In addition, a nomogram was established to predict the survival time of CC patients. Finally, the relationship with immunotherapy responses was investigated. Surprisingly, there was a significant correlation between the m6A-LPS and the tumor-infiltrating immune cells. The m6A-LPS has potential as a predictor of ICI treatment response and chemosensitivity in CC patients.

Chang et al. (2021) identified a positive feedback loop “ITGB1-DT/ITGB1/Wnt/β-catenin/MYC” among the 14 hub lncRNAs, significantly promoting the proliferation, migration, and invasive ability of lung adenocarcinoma cells. The enhanced expression of *ITGB1-DT*, an oncogenic lncRNA, has been linked to the poor OS and disease-free survival (Chang et al., 2021). *NCBP2-AS1* has been implicated in a model of colon adenocarcinoma recurrence prognosis based on competing endogenous RNAs (Jin et al., 2020). In renal cell carcinoma, *AP006621.2* has been identified as a redox-related prognostic factor (Qi-Dong et al., 2020). In bladder cancer, *SNHG26* was discovered to be an epithelial-mesenchymal transition-related prognostic factor (Tong et al., 2021). In addition, *AC073896.3* and *TNFRSF10A-AS1* have been used as components of an autophagy-related lncRNA signature to improve colorectal cancer prognosis (Wei et al., 2020; Zhou et al., 2020). Meanwhile, AC073896.3 and TNFRSF10A-AS1 are prognostic favorable factors for colon cancer, which is consistent with our study (Wei et al., 2020; Zhou et al., 2020). AL137782.1 and AC073896.3 are protective factors for colon cancer, which is consistent with our study (Zhang et al., 2021b). Recently, *AC099850.3* was proven to be involved in the migration and proliferation of hepatocellular carcinoma by regulating the expression of cell-cycle-related molecules (Wu et al., 2021). In addition, *AC099850.3* was highly expressed in HCC and squamous cell carcinoma of the tongue and increased the predicted value of the signature through coexpression and the ceRNA mechanism (Zhou et al., 2019; Jia et al., 2020; Wu et al., 2020; Jiang et al., 2021). However, there have been few reports on the other lncRNAs.

Compared with the similar study by Chen S (Chen et al., 2021), our model has a better predictive value for OS of cancer patients at 1-, 3-, 5-year. Furthermore, we also comprehensively investigated the relationship between the model and consensus cluster, tumor infiltration immune cells, immunosuppressed biomarkers, somatic variants, and chemotherapeutic drug efficacy. Inevitably, there were several limitations to this study. As a retrospective study, this study demonstrated a certain degree of heterogeneity among patients, hence, future validation studies are required. In addition, the m6A-LPS, as well as its correlation with TME, did not undergo external verification because of insufficient data from other independent cohorts. Therefore, several methods were utilized to verify this novel prognostic signature. Finally, the data of TCGA released publicly was mainly mined and analyzed as this study required further external validation in multicenter cohorts.

## Conclusion

This is the first study that comprehensively identified and systematically analyzed the expression data of m6A-related lncRNAs in CC in the TCGA database. The m6A-LPS, which is based on 14 m6A-related lncRNAs, has been revealed to be a novel potential and promising biomarker for evaluating the prognosis of CC patients. The survival rate, clinical features, tumor infiltration immune cells, immunosuppressed biomarkers, and chemotherapeutic drug efficacy were all re-evaluated. This study revealed that the risk signature is a promising predictive indicator that may provide more accurate clinical applications in CC therapeutics and enable effective therapy strategies for clinicians.

## Data Availability

The original contributions presented in the study are included in the article/Supplementary Material, further inquiries can be directed to the corresponding author.

## References

[B1] Aran D. (2020). Cell-type enrichment analysis of bulk transcriptomes using xCell. Methods Mol. Biol. 2120, 263–276. 10.1007/978-1-0716-0327-7_19 32124326

[B2] Aran D. Hu Z. Butte A. J. (2017). xCell: digitally portraying the tissue cellular heterogeneity landscape. Genome Biol. 18, 220. 10.1186/s13059-017-1349-1 29141660 PMC5688663

[B3] Chang R. Xiao X. Fu Y. Zhang C. Zhu X. Gao Y. (2021). ITGB1-DT facilitates lung adenocarcinoma progression via forming a positive feedback loop with ITGB1/wnt/β-catenin/MYC. Front. Cell Dev. Biol. 9, 631259. 10.3389/fcell.2021.631259 33763420 PMC7982827

[B4] Chen B. Khodadoust M. S. Liu C. L. Newman A. M. Alizadeh A. A. (2018). Profiling tumor infiltrating immune cells with CIBERSORT. Methods Mol. Biol. 1711, 243–259. 10.1007/978-1-4939-7493-1_12 29344893 PMC5895181

[B5] Chen S. Li X. Guo L. Zhang J. Li L. Wang X. (2021). Characterization of the m6A-related lncRNA signature in predicting prognosis and immune response in patients with colon cancer. J. B.U.ON, official J. Balkan Union Oncol. 26, 1931–1941. 34761602

[B6] Dienstmann R. Villacampa G. Sveen A. Mason M. J. Niedzwiecki D. Nesbakken A. (2019). Relative contribution of clinicopathological variables, genomic markers, transcriptomic subtyping and microenvironment features for outcome prediction in stage II/III colorectal cancer. Ann. Oncol. 30, 1622–1629. 10.1093/annonc/mdz287 31504112 PMC6857614

[B7] Feng Z. Gao H. Feng T. (2021). Immune infiltrates of m^6^A RNA methylation-related lncRNAs and identification of PD-L1 in patients with primary head and neck squamous cell carcinoma. Front. Cell Dev. Biol. 9, 672248. 10.3389/fcell.2021.672248 34178999 PMC8220827

[B8] Jia Y. Chen Y. Liu J. (2020). Prognosis-predictive signature and nomogram based on autophagy-related long non-coding RNAs for hepatocellular carcinoma. Front. Genet. 11, 608668. 10.3389/fgene.2020.608668 33424932 PMC7793718

[B9] Jiang Q. Xue D. Shi F. Qiu J. (2021). Prognostic significance of an autophagy-related long non-coding RNA signature in patients with oral and oropharyngeal squamous cell carcinoma. Oncol. Lett. 21, 29. 10.3892/ol.2020.12290 33240435 PMC7681235

[B10] Jin L. Liu T. Meng F. Tai J. (2020). Prognosis prediction model based on competing endogenous RNAs for recurrence of colon adenocarcinoma. BMC cancer 20, 968. 10.1186/s12885-020-07163-y 33028275 PMC7541229

[B11] Johnsson P. Lipovich L. Grander D. Morris K. V. (2014). Evolutionary conservation of long non-coding RNAs; sequence, structure, function. Biochim. Biophys. Acta 1840, 1063–1071. 10.1016/j.bbagen.2013.10.035 24184936 PMC3909678

[B12] Kanehisa M. Furumichi M. Tanabe M. Sato Y. Morishima K. (2017). Kegg: New perspectives on genomes, pathways, diseases and drugs. Nucleic Acids Res. 45, D353–D361. 10.1093/nar/gkw1092 27899662 PMC5210567

[B13] Lan Y. Liu B. Guo H. (2021). The role of M^6^A modification in the regulation of tumor-related lncRNAs. Mol. Ther. Nucleic acids 24, 768–779. 10.1016/j.omtn.2021.04.002 33996258 PMC8094576

[B14] Li T. Fan J. Wang B. Traugh N. Chen Q. Liu J. S. (2017). Timer: A web server for comprehensive analysis of tumor-infiltrating immune cells. Cancer Res. 77, e108–e110. 10.1158/0008-5472.Can-17-0307 29092952 PMC6042652

[B15] Lu G. Zhang Y. (2020). Long non-coding RNA ATB promotes human non-small cell lung cancer proliferation and metastasis by suppressing miR-141-3p. PLoS ONE 15, e0229118. 10.1371/journal.pone.0229118 32092085 PMC7039450

[B16] Ma S. Chen C. Ji X. Liu J. Zhou Q. Wang G. (2019). The interplay between m6A RNA methylation and noncoding RNA in cancer. J. Hematol. Oncol. 12, 121. 10.1186/s13045-019-0805-7 31757221 PMC6874823

[B17] Ma Z. Huang H. Xu Y. He X. Wang J. Hui B. (2017). Current advances of long non-coding RNA highly upregulated in liver cancer in human tumors. Onco Targets Ther. 10, 4711–4717. 10.2147/OTT.S136915 29026319 PMC5626378

[B18] Ni W. Yao S. Zhou Y. Liu Y. Huang P. Zhou A. (2019). Long noncoding RNA GAS5 inhibits progression of colorectal cancer by interacting with and triggering YAP phosphorylation and degradation and is negatively regulated by the m6A reader YTHDF3. Mol. cancer 18, 143. 10.1186/s12943-019-1079-y 31619268 PMC6794841

[B19] Plattner C. Finotello F. Rieder D. (2020). Deconvoluting tumor-infiltrating immune cells from RNA-seq data using quanTIseq. Methods Enzymol. 636, 261–285. 10.1016/bs.mie.2019.05.056 32178821

[B20] Qi-Dong X. Yang X. Lu J. Liu C. Sun J. Li C. (2020). Development and validation of a nine-redox-related long noncoding RNA signature in renal clear cell carcinoma. Oxidative Med. Cell. Longev. 2020, 6634247. 10.1155/2020/6634247 PMC7781722 33425212

[B21] Racle J. de Jonge K. Baumgaertner P. Speiser D. E. Gfeller D. (2017). Simultaneous enumeration of cancer and immune cell types from bulk tumor gene expression data. Elife 6, e26476. 10.7554/eLife.26476 29130882 PMC5718706

[B22] Subramanian A. Tamayo P. Mootha V. K. Mukherjee S. Ebert B. L. Gillette M. A. (2005). Gene set enrichment analysis: A knowledge-based approach for interpreting genome-wide expression profiles. Proc. Natl. Acad. Sci. U. S. A. 102, 15545–15550. 10.1073/pnas.0506580102 16199517 PMC1239896

[B23] Tamminga M. Hiltermann T. J. N. Schuuring E. Timens W. Fehrmann R. S. Groen H. J. (2020). Immune microenvironment composition in non-small cell lung cancer and its association with survival. Clin. Transl. Immunol. 9, e1142. 10.1002/cti2.1142 PMC7291326 32547744

[B24] Tian S. Lai J. Yu T. Li Q. Chen Q. (2020). Regulation of gene expression associated with the N6-methyladenosine (m6A) enzyme System and its significance in cancer. Front. Oncol. 10, 623634. 10.3389/fonc.2020.623634 33552994 PMC7859513

[B25] Tong H. Li T. Gao S. Yin H. Cao H. He W. (2021). An epithelial-mesenchymal transition-related long noncoding RNA signature correlates with the prognosis and progression in patients with bladder cancer. Biosci. Rep. 41, BSR20203944. 10.1042/bsr20203944 33289830 PMC7786330

[B26] Wang B. Lu Z. Huang Y. Lin T. (2020). Prognostic impact of lncRNA-ATB expression in malignant solid tumors: A meta-analysis. Pathology Res. Pract. 216, 152897. 10.1016/j.prp.2020.152897 32146004

[B27] Wang H. Meng Q. Ma B. (2021). Characterization of the prognostic m6A-related lncRNA signature in gastric cancer. Front. Oncol. 11, 630260. 10.3389/fonc.2021.630260 33928026 PMC8076577

[B46] Wang Y. Lin K. Xu T. Wang L. Fu L. Zhang G. (2021). Development and validation of prognostic model based on the analysis of autophagy-related genes in colon cancer. Aging. 13(14), 19028-19047. 34315829 10.18632/aging.203352 PMC8351728

[B47] Wang Y. Fu L. Lu T. Zhang G. Zhang J. Zhao Y. (2021). Clinicopathological and Prognostic Significance of Long Non-coding RNA MIAT in Human Cancers: A Review and Meta-Analysis. Frontiers in genetics. 12, 729768. 34659354 10.3389/fgene.2021.729768 PMC8514773

[B28] Wei J. Ge X. Tang Y. Qian Y. Lu W. Jiang K. (2020). An autophagy-related long noncoding RNA signature contributes to poor prognosis in colorectal cancer. J. Oncol. 2020, 4728947. 10.1155/2020/4728947 33149738 PMC7603611

[B29] Wilkerson M. Hayes D. ConsensusClusterPlus (2010). ConsensusClusterPlus: A class discovery tool with confidence assessments and item tracking. Bioinforma. Oxf. Engl. 26, 1572–1573. 10.1093/bioinformatics/btq170 PMC2881355 20427518

[B30] Wilusz J. E. Sunwoo H. Spector D. L. (2009). Long noncoding RNAs: Functional surprises from the RNA world. Genes Dev. 23, 1494–1504. 10.1101/gad.1800909 19571179 PMC3152381

[B31] Wu F. Wei H. Liu G. Zhang Y. (2021). Bioinformatics profiling of five immune-related lncRNAs for a prognostic model of hepatocellular carcinoma. Front. Oncol. 11, 667904. 10.3389/fonc.2021.667904 34123835 PMC8195283

[B32] Wu H. Liu T. Qi J. Qin C. Zhu Q. (2020). Four autophagy-related lncRNAs predict the prognosis of HCC through coexpression and ceRNA mechanism. BioMed Res. Int. 2020, 3801748. 10.1155/2020/3801748 33102579 PMC7568797

[B33] Wu Y. Yang X. Chen Z. Tian L. Jiang G. Chen F. (2019). m6A-induced lncRNA RP11 triggers the dissemination of colorectal cancer cells via upregulation of Zeb1. Mol. cancer 18, 87. 10.1186/s12943-019-1014-2 30979372 PMC6461827

[B45] Xiaoyong H. Rangyin Z. Guangming Z. Yajun J. Yongfeng W. Da W. (2022). Association of Retinol and Carotenoids Content in Diet and Serum With Risk for Colorectal Cancer: A Meta-Analysis. Frontiers in nutrition 9, 918777. 10.3389/fnut.2022.918777 35845801 PMC9280435

[B34] Xavier R. J. Podolsky D. K. (2007). Unravelling the pathogenesis of inflammatory bowel disease. Nature 448, 427–434. 10.1038/nature06005 17653185

[B35] Xu F. Huang X. Li Y. Chen Y. Lin L. (2021). m^6^A-related lncRNAs are potential biomarkers for predicting prognoses and immune responses in patients with LUAD. Mol. Ther. Nucleic acids 24, 780–791. 10.1016/j.omtn.2021.04.003 33996259 PMC8094594

[B36] Xu R. Pang G. Zhao Q. Yang L. Chen S. Jiang L. (2021). The momentous role of N6-methyladenosine in lung cancer. J. Cell. physiology 236, 3244–3256. 10.1002/jcp.30136 33135190

[B48] Y W. X J. D Z. Y Z. X H. L Z. (2022). LncRNA DUXAP8 as a prognostic biomarker for various cancers: A meta-analysis and bioinformatics analysis. Frontiers in genetics 13, 907774. 36046244 10.3389/fgene.2022.907774 PMC9420988

[B37] Yang X. Zhang S. He C. Xue P. Zhang L. He Z. (2020). METTL14 suppresses proliferation and metastasis of colorectal cancer by down-regulating oncogenic long non-coding RNA XIST. Mol. cancer 19, 46. 10.1186/s12943-020-1146-4 32111213 PMC7047419

[B38] Zaccara S. Ries R. Jaffrey S. (2019). Reading, writing and erasing mRNA methylation. Nat. Rev. Mol. Cell Biol. 20, 608–624. 10.1038/s41580-019-0168-5 31520073

[B39] Zhang H. Li R. Cao Y. Gu Y. Lin C. Liu X. (2020). Poor clinical outcomes and immunoevasive contexture in intratumoral IL-10-producing macrophages enriched gastric cancer patients. Ann. Surg. 275, e626–e635. 10.1097/sla.0000000000004037 32541216

[B40] Zhang J. Zhang X. Piao C. Bi J. Zhang Z. Li Z. (2019). A long non-coding RNA signature to improve prognostic prediction in clear cell renal cell carcinoma. Biomed. Pharmacother. = Biomedecine Pharmacother. 118, 109079. 10.1016/j.biopha.2019.109079 31351427

[B41] Zhang L. Wan Y. Zhang Z. Jiang Y. Gu Z. Ma X. (2021). IGF2BP1 overexpression stabilizes PEG10 mRNA in an m6A-dependent manner and promotes endometrial cancer progression. Theranostics 11, 1100–1114. 10.7150/thno.49345 33391523 PMC7738899

[B42] Zhang P. Liu G. Lu L. (2021). N6-Methylandenosine-Related lncRNA signature is a novel biomarkers of prognosis and immune response in colon adenocarcinoma patients. Front. Cell Dev. Biol. 9, 703629. 10.3389/fcell.2021.703629 34336856 PMC8321625

[B43] Zhou R. Zhang E. Sun Q. Ye Z. Liu J. Zhou D. (2019). Integrated analysis of lncRNA-miRNA-mRNA ceRNA network in squamous cell carcinoma of tongue. BMC cancer 19, 779. 10.1186/s12885-019-5983-8 31391008 PMC6686570

[B44] Zhou W. Zhang S. Li H. Cai Z. Tang S. Chen L. (2020). Development of prognostic indicator based on autophagy-related lncRNA analysis in colon adenocarcinoma. BioMed Res. Int. 2020, 9807918. 10.1155/2020/9807918 32964050 PMC7486634

