# Supplementary material for: Constructing a novel signature and predicting the immune landscape of colon cancer using N6-methylandenosine-related lncRNAs
Source: Front Genet. 2023 Jun 16;14:906346. doi: 10.3389/fgene.2023.906346 (PMC10313068; doi:10.3389/fgene.2023.906346)
Supplement: Supplementary file 1 [file Table1.DOCX]

Supplemental Information

Supplementary Table 1. A total of the m6A-Related prognostic lncRNAs were identified by co-expression analysis (submitted as a separate Excel file).

Supplementary Table 2. Univariate Cox regression analysis of the 38 m6A-Related prognostic lncRNAs.

Supplementary Table 3. LASSO regression for 14 m6A-related prognostic lncRNAs in the m6A- LPS.

Supplementary Table 4. The detail values of correlations between the m6A-related prognostic lncRNAs and clinical features.

Supplementary Table 5. The detail comparison results of correlation ship between tumour infiltrating immune cells and risk sore.

Supplementary Figure 1. Consensus clustering matrix for k = 2 to 9.

Supplementary Figure 2. The representative results of the evaluation of tumor infiltrating immune cells with clusters.

Supplementary Figure 3. The bar plot of 14 prognostic lncRNAs in normal and tumor tissues.

Supplementary Figure 4. Kaplan–Meier curves for prognostic value of the 14 prognostic lncRNAs in the m6A- LPS.

Supplementary Figure 5. Figure 7. Kaplan–Meier survival curve of different factors for the patients with CC in TCGA dataset. (A) age, (B) gender, (C) stage (D) T, (E) M, (F) N.

Supplementary Figure 6. The representative results of the evaluation of tumor infiltrating immune cells with risk assessment model.

**Supplementary Table 1. A total of the m6A-Related prognostic lncRNAs were identified by co-expression analysis (submitted as a separate Excel file).**

Table S2. Univariate Cox regression analysis of the 38 m6A-Related prognostic lncRNAs.

| gene | HR | HR.95L | HR.95H | pvalue |
| --- | --- | --- | --- | --- |
| AKR1C1 | 1.002316 | 1.00058 | 1.004055 | 0.00889 |
| AKR1C3 | 1.003568 | 1.001635 | 1.005505 | 0.000293 |
| CARS1 | 1.07825 | 1.021505 | 1.138148 | 0.006308 |
| CBS | 0.782145 | 0.617541 | 0.990624 | 0.04154 |
| CISD1 | 1.049558 | 1.01839 | 1.08168 | 0.001662 |
| FANCD2 | 1.33217 | 1.145022 | 1.549906 | 0.000205 |
| GSS | 1.016716 | 1.003637 | 1.029967 | 0.012092 |
| SLC7A11 | 1.075818 | 1.02066 | 1.133956 | 0.006499 |
| FDFT1 | 1.012548 | 1.001381 | 1.023839 | 0.027539 |
| TFRC | 1.018317 | 1.000717 | 1.036227 | 0.041299 |
| AIFM2 | 1.042788 | 1.012992 | 1.073461 | 0.004616 |
| FTH1 | 1.001304 | 1.000476 | 1.002132 | 0.002019 |
| STEAP3 | 0.98964 | 0.979626 | 0.999757 | 0.044779 |
| ACSL3 | 1.035678 | 1.014728 | 1.057061 | 0.000773 |
| ACACA | 1.133018 | 1.059853 | 1.211232 | 0.000246 |
| SQLE | 1.008577 | 1.001409 | 1.015796 | 0.018937 |
| KEAP1 | 1.028657 | 1.006795 | 1.050994 | 0.009941 |
| NQO1 | 1.002071 | 1.00094 | 1.003203 | 0.000331 |
| ABCC1 | 1.073045 | 1.036112 | 1.111294 | 7.98E-05 |
| SLC1A5 | 1.012409 | 1.006083 | 1.018775 | 0.000115 |
| G6PD | 1.012815 | 1.008713 | 1.016934 | 7.79E-10 |
| PGD | 1.008085 | 1.004607 | 1.011575 | 4.95E-06 |
| HMOX1 | 1.002532 | 1.001082 | 1.003984 | 0.000616 |

Supplementary Table 3. LASSO regression for 14 m6A-related prognostic lncRNAs in the m6A- LPS.

| Gene | Coef |
| --- | --- |
| TNFRSF10A-AS1 | -0.02102 |
| AC245041.1 | 0.259351 |
| AL513550.1 | 0.19614 |
| UTAT33 | 0.21851 |
| SNHG26 | 0.075165 |
| AC092944.1 | 1.187719 |
| ITGB1-DT | 0.433276 |
| AL138921.1 | 1.296707 |
| AC099850.3 | -0.04355 |
| NCBP2-AS1 | 0.034475 |
| AL137782.1 | -0.53815 |
| AC073896.3 | -0.20653 |
| AP006621.2 | 0.09041 |
| AC147651.1 | 0.299669 |

**Supplementary Table 4. The detail values of correlations between the m6A-related prognostic lncRNAs and clinical features.**

| id | age | gender | stage | T | M | N |
| --- | --- | --- | --- | --- | --- | --- |
| TNFRSF10A-AS1 | 16152(0.112) | 19575(0.414) | 16.635(8.402e-04) | 3.897(0.273) | 10568(0.436) | 14.864(5.92e-04) |
| AC245041.1 | 20398(0.010) | 18901(0.824) | 1.067(0.785) | 2.05(0.562) | 9310(0.391) | 4.435(0.109) |
| AL513550.1 | 18373(0.633) | 18246(0.696) | 1.918(0.590) | 5.151(0.161) | 9304(0.426) | 2.167(0.338) |
| UTAT33 | 17045(0.448) | 17827(0.440) | 0.623(0.891) | 1.285(0.733) | 9354(0.463) | 1.163(0.559) |
| SNHG26 | 17077.5(0.467) | 19619.5(0.391) | 0.471(0.925) | 4.239(0.237) | 10406.5(0.564) | 10.5(0.005) |
| AC092944.1 | 18008(0.891) | 18222.5(0.680) | 0.899(0.826) | 1.829(0.609) | 9885(0.943) | 1.215(0.545) |
| ITGB1-DT | 18079(0.839) | 18298(0.731) | 2.988(0.394) | 3.979(0.264) | 9134(0.313) | 5.735(0.057) |
| AL138921.1 | 16991(0.419) | 18535.5(0.899) | 1.485(0.686) | 10.529(0.015) | 9398.5(0.497) | 4.936(0.085) |
| AC099850.3 | 18017(0.884) | 17216(0.184) | 4.812(0.186) | 5.715(0.126) | 10931(0.218) | 6.65(0.036) |
| NCBP2-AS1 | 18579(0.504) | 19028.5(0.749) | 2.054(0.561) | 4.057(0.255) | 9394(0.494) | 1.159(0.560) |
| AL137782.1 | 16931(0.388) | 18229(0.685) | 5.919(0.116) | 1.416(0.702) | 8086(0.021) | 2.206(0.332) |
| AC073896.3 | 17343(0.631) | 19803(0.305) | 2.424(0.489) | 0.285(0.963) | 10499(0.488) | 1.928(0.381) |
| AP006621.2 | 16381.5(0.169) | 17396.5(0.244) | 2.688(0.442) | 4.245(0.236) | 8853.5(0.174) | 3.516(0.172) |
| AC147651.1 | 17652(0.841) | 18328(0.743) | 8.558(0.036) | 5.416(0.144) | 8678(0.102) | 4.772(0.092) |
| riskScore | 18069(0.846) | 18696(0.986) | 11.589(0.009) | 17.681(5.117e-04) | 8458(0.064) | 16.119(3.161e-04) |

Supplementary Table 5. The detail comparison results of correlation ship between tumour infiltrating immune cells and risk sore.

| immune | cor | pvalue |
| --- | --- | --- |
| T cell CD4+_TIMER | 0.169849 | 0.000320322 |
| T cell CD8+_TIMER | -0.12899 | 0.006374054 |
| T cell CD8+_CIBERSORT | 0.122892 | 0.009379766 |
| T cell CD4+ memory resting_CIBERSORT | -0.0929 | 0.049912301 |
| T cell CD4+ memory activated_CIBERSORT | -0.17557 | 0.000194273 |
| T cell regulatory (Tregs)_CIBERSORT | 0.203645 | 1.46E-05 |
| Mast cell resting_CIBERSORT | -0.23445 | 5.52E-07 |
| Neutrophil_CIBERSORT | -0.14133 | 0.002777592 |
| T cell CD8+_CIBERSORT-ABS | 0.096519 | 0.041610043 |
| T cell CD4+ memory activated_CIBERSORT-ABS | -0.16688 | 0.000401196 |
| T cell regulatory (Tregs)_CIBERSORT-ABS | 0.207098 | 1.04E-05 |
| Mast cell resting_CIBERSORT-ABS | -0.21465 | 4.79E-06 |
| Neutrophil_CIBERSORT-ABS | -0.12408 | 0.00871186 |
| B cell_QUANTISEQ | 0.174863 | 0.00020632 |
| Macrophage M1_QUANTISEQ | -0.14628 | 0.001971041 |
| Macrophage M2_QUANTISEQ | 0.210276 | 7.52E-06 |
| Neutrophil_QUANTISEQ | -0.12306 | 0.009316459 |
| T cell_MCPCOUNTER | 0.120882 | 0.010653783 |
| T cell CD4+ memory_XCELL | -0.20475 | 1.31E-05 |
| T cell CD4+ naive_XCELL | 0.144615 | 0.002201372 |
| T cell CD4+ central memory_XCELL | 0.244856 | 1.64E-07 |
| T cell CD4+ effector memory_XCELL | 0.095596 | 0.043609676 |
| Common lymphoid progenitor_XCELL | -0.22499 | 1.69E-06 |
| Hematopoietic stem cell_XCELL | 0.121002 | 0.010538465 |
| Neutrophil_XCELL | -0.15187 | 0.001295473 |
| Plasmacytoid dendritic cell_XCELL | -0.09541 | 0.04402868 |
| T cell gamma delta_XCELL | -0.13531 | 0.004199086 |
| T cell CD4+ Th2_XCELL | -0.27218 | 5.14E-09 |
| T cell CD4+_EPIC | 0.096896 | 0.040848749 |
| uncharacterized cell_EPIC | -0.13364 | 0.00472611 |


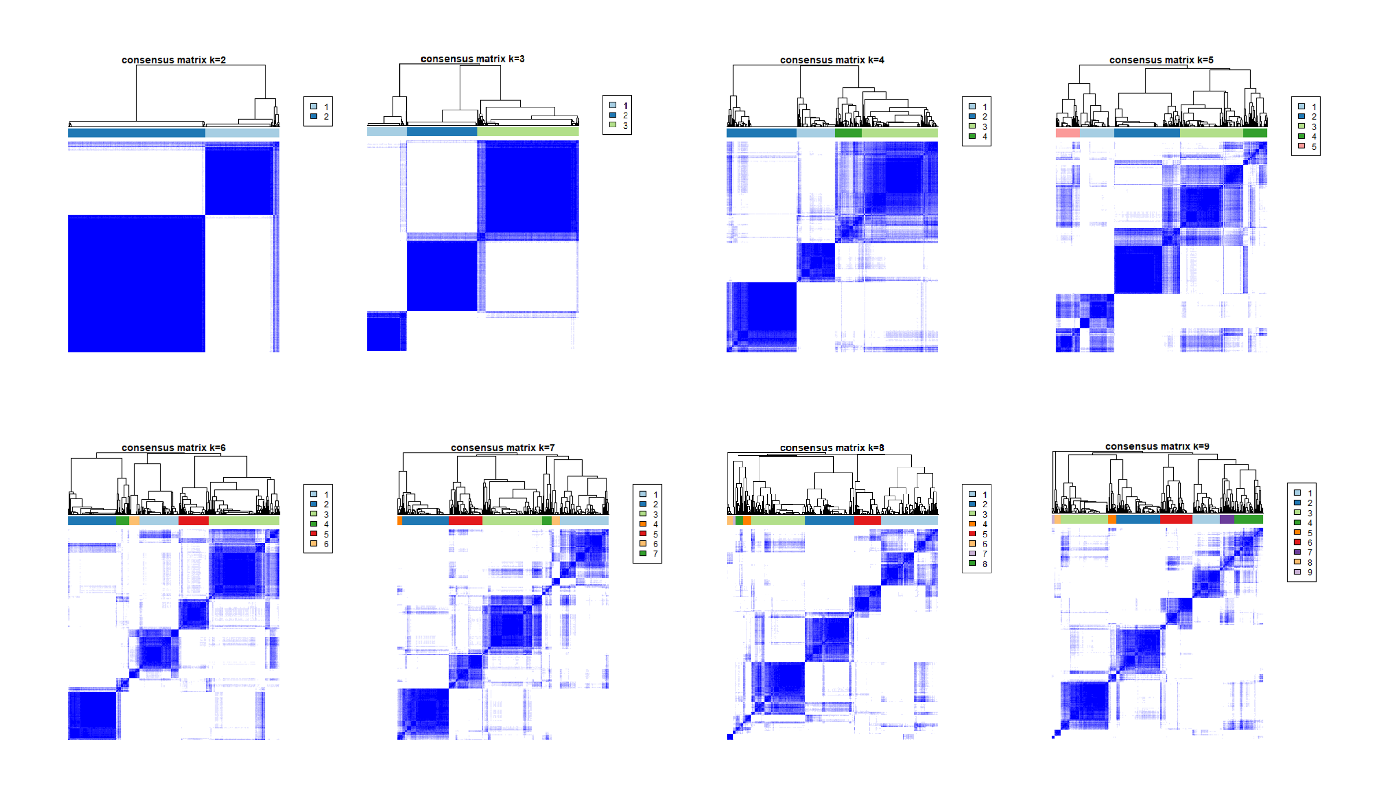


Supplementary Figure 1. Consensus clustering matrix for k = 2 to 9.


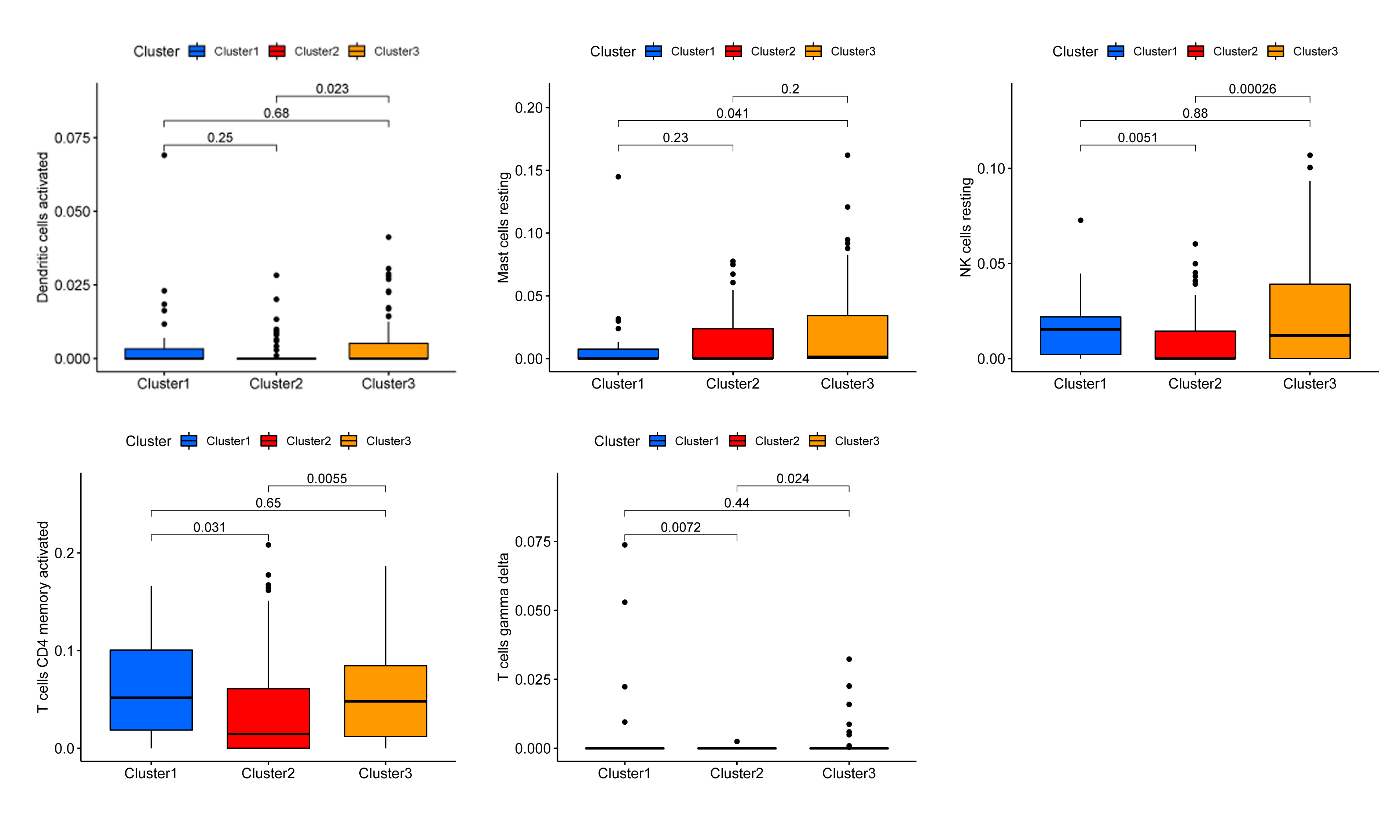


Supplementary Figure 2. The representative results of the evaluation of tumor infiltrating immune cells with clusters.


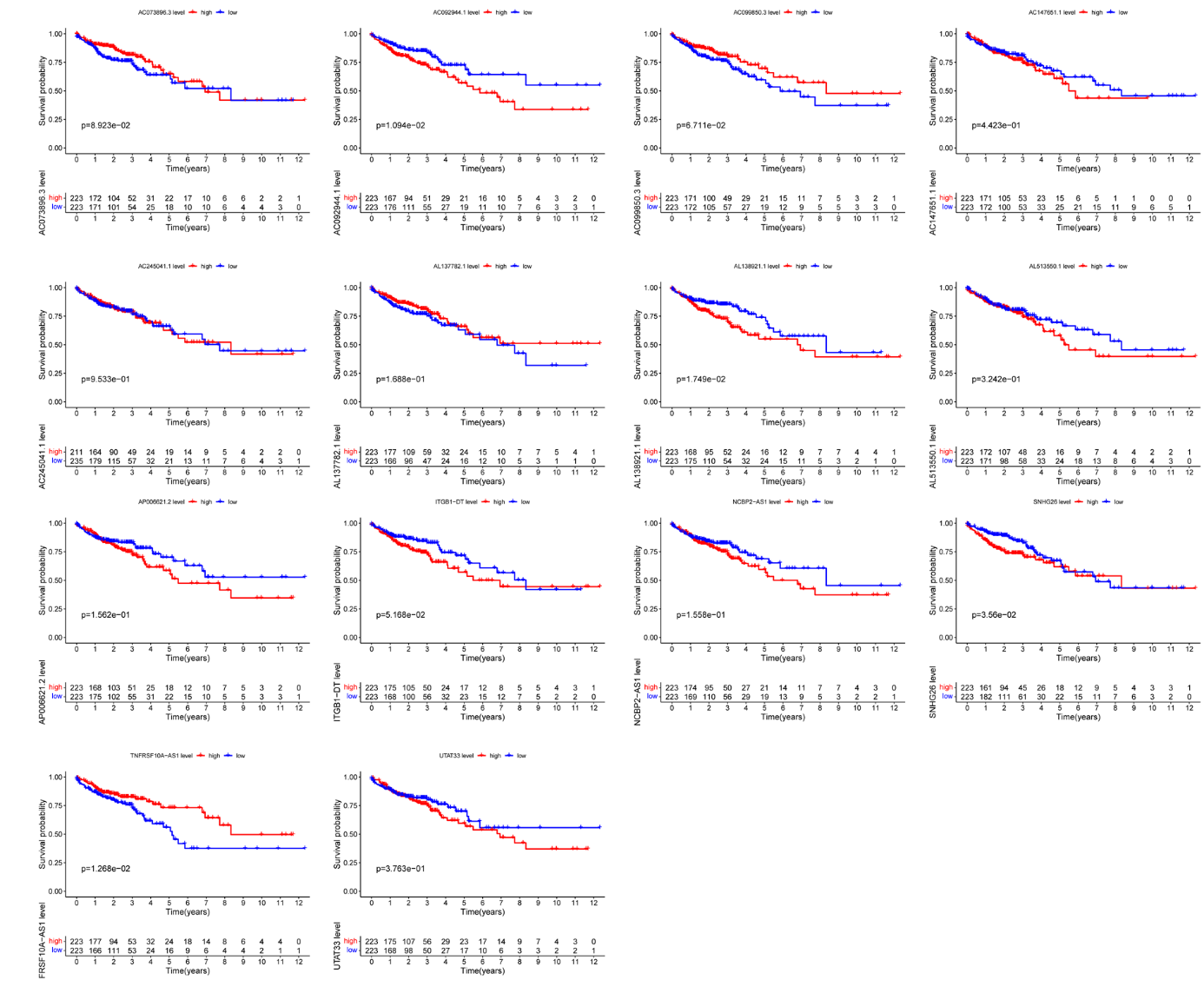


Supplementary Figure 3. Kaplan–Meier curves for prognostic value of the 14 prognostic lncRNAs in the m6A- LPS.


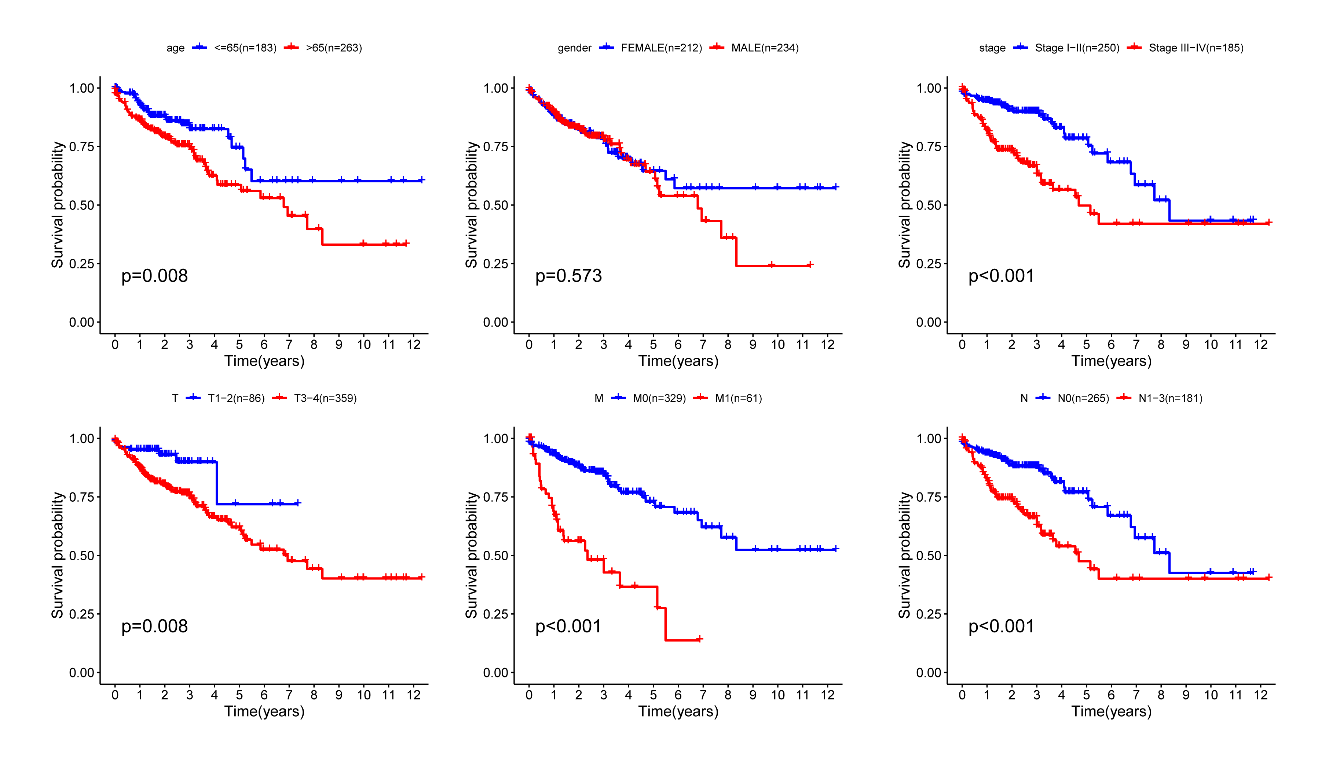


Supplementary Figure 4. Figure 7. Kaplan–Meier survival curve of different factors for the patients with CC in TCGA dataset. (A) age, (B) gender, (C) stage (D) T, (E) M, (F) N.


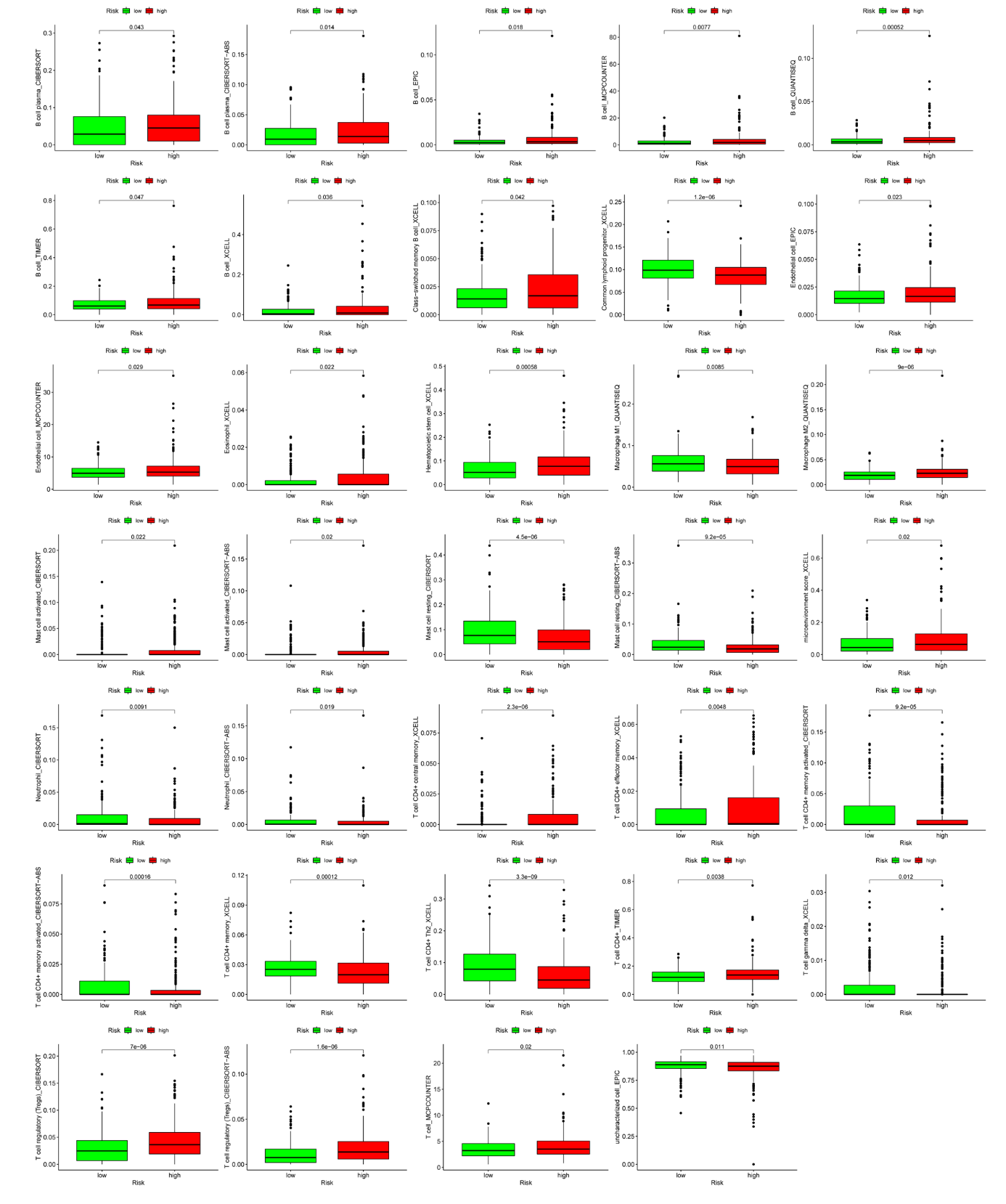


Supplementary Figure 5. The representative results of the evaluation of tumor infiltrating immune cells with risk assessment model.
